# Chloroplast stress caused by maltose hyperaccumulation activates chlorophagy via the core autophagy machinery

**DOI:** 10.1093/plphys/kiag271

**Published:** 2026-05-08

**Authors:** Sakuya Nakamura, Mayumi Wakazaki, Mayuko Sato, Kiminori Toyooka, Atsushi J Nagano, Hiroyuki Ishida, Shinya Hagihara, Masanori Izumi

**Affiliations:** RIKEN Center for Sustainable Resource Science (CSRS), Wako 351-0198, Japan; RIKEN Center for Sustainable Resource Science (CSRS), Wako 351-0198, Japan; RIKEN Center for Sustainable Resource Science (CSRS), Wako 351-0198, Japan; RIKEN Center for Sustainable Resource Science (CSRS), Wako 351-0198, Japan; Bioscience and Biotechnology Center, Nagoya University, Nagoya 464-8601, Japan; Institute for Advanced Biosciences, Keio University, Tsuruoka 997-0017, Japan; Graduate School of Agricultural Science, Tohoku University, Sendai 980-8572, Japan; RIKEN Center for Sustainable Resource Science (CSRS), Wako 351-0198, Japan; RIKEN Center for Sustainable Resource Science (CSRS), Wako 351-0198, Japan

## Abstract

Chlorophagy is an autophagy pathway that delivers chloroplast components into the vacuole for degradation, thus eliminating damaged chloroplasts. Chloroplast degradation is observed in Arabidopsis (*Arabidopsis thaliana*) mutants of MALTOSE-EXCESS 1 (MEX1), a maltose exporter in the chloroplast inner envelope membrane. However, whether autophagy is involved in the *mex1* phenotypes is unknown. To extend our understanding of the signals that emanate from damaged chloroplasts and activate chlorophagy, we investigated how *mex1* chloroplasts are degraded. Chlorotic mature leaves caused by maltose hyperaccumulation in *mex1* plants contained swollen chloroplasts in the cytoplasm and degrading chloroplasts in the vacuole, together with heightened expression of autophagy-related (*ATG*) genes. The vacuolar degradation of *mex1* chloroplasts required the core ATG proteins ATG7 and ATG10. ATG8-labeled structures accumulated on the surfaces of swollen *mex1* chloroplasts. These findings indicate that maltose hyperaccumulation triggers chlorophagy via the core autophagy machinery. Notably, phenotypic analysis of *mex1 atg* double mutant plants suggested that excess chlorophagy aggravates the chlorosis seen in *mex1* leaves. Transcriptome deep sequencing indicated that maltose-excess stress shares a similar transcriptomic response with high-light stress, which also triggers chlorophagy. Therefore, the signals inducing chlorophagy may be highly stimulated in *mex1* leaves, making *mex1* mutants effective tools for chlorophagy research.

## Teaser

Excess maltose accumulation in chloroplasts stimulates signals that induce chlorophagy, a core autophagy machinery-dependent process degrading entire chloroplasts.

## Introduction

Chloroplasts are organelles that serve as the sites of energy production for plant growth via photosynthesis. Chloroplasts are normally exposed to oxidative damage, as photosynthetic reactions are a principal source of reactive oxygen species (ROS) in the cells of green tissues (Takahashi and Badger 2011; Li and Kim 2022; Woodson 2022). The quality of the chloroplast population must therefore be controlled to support healthy plant growth. We previously revealed that chloroplasts damaged in response to strong light are eliminated into the vacuolar lumen via an autophagy program termed chlorophagy (Izumi et al. 2017; Nakamura et al. 2018).

Autophagy is a conserved intracellular set of reactions in eukaryotes that transports cytoplasmic components into the vacuole or lysosome for degradation. Autophagy is critical for eliminating damaged organelles and controlling cellular quality in various organisms (Vargas et al. 2023). One well-characterized form of autophagy is macroautophagy, which takes place through the formation of a cargo-sequestering, double membrane–bound structure termed the autophagosome (Nakatogawa 2020). Core autophagy-related proteins encoded by autophagy-related genes (in Arabidopsis [*Arabidopsis thaliana*], *AUTOPHAGY1* (*ATG1*)*–10*, *ATG12–14*, *ATG16*, and *ATG18*) help form autophagosomes and are highly conserved among plants, yeasts, and animals (Yoshimoto and Ohsumi 2018; Nakatogawa 2020; Nakamura et al. 2021a). Of these, the ubiquitin-like protein ATG8, conjugated to phosphatidylethanolamine (PE), is a structural component of the autophagosomal membrane (Ichimura et al. 2000), making ATG8 a well-characterized marker of autophagosome-associated membranes. ATG3–5, ATG7, ATG10, ATG12, and ATG16 mediate the production of the ATG8–PE conjugate (Nakatogawa 2020).

Microautophagy is another form of autophagy during which substrates are sequestered by the membranes of the vacuole, lysosomes, or endosomes without being encapsulated by the autophagosome (Sakai et al. 2025). In various organisms including yeasts, plants, and mammals, core ATG protein-dependent and protein-independent types of microautophagy have been identified (Wang et al. 2023; Sakai et al. 2025). During chlorophagy in Arabidopsis leaves exposed to strong light, flattened structures containing ATG8 accumulate on parts of the surfaces of damaged chloroplasts, which are then sequestered by the vacuolar membrane (Nakamura et al. 2018). Neither these phenomena nor the vacuolar accumulation of chloroplasts undergoing degradation occurs in mutants of *ATG5* or *ATG7* (Izumi et al. 2017; Nakamura et al. 2018). These findings reveal that a type of microautophagy that requires core ATG functions mediates the selective elimination of damaged chloroplasts caused by exposure to strong light. Therefore, elucidating the mechanisms regulating chlorophagy would expand our understanding of the molecular mechanisms underlying microautophagy and plant adaptive responses to photooxidative damage. However, how chlorophagy is regulated remains poorly understood.

To overcome this limitation, we aimed to identify chloroplast stress besides strong light damage that could trigger chlorophagy. A previous study revealed the occurrence of chloroplast degradation in the leaves of a mutant of the chloroplast inner envelope–bound transporter MALTOSE-EXCESS 1 (MEX1; Stettler et al. 2009), a maltose exporter that directs maltose to the cytoplasm from chloroplasts (Niittylä et al. 2004; Lu et al. 2006). Arabidopsis leaves accumulate some photosynthetic assimilates as starch granules in their chloroplasts during the day via starch-producing enzymes including PHOSPHOGLUCOMUTASE (PGM; Caspar et al. 1985). At night, starch granules are broken down by starch-degrading enzymes including GWD (α-glucan, water dikinase; also known as STARCH-EXCESS 1 [SEX1]) (Caspar et al. 1991; Yu et al. 2001). The major degradation product of starch is maltose, which is exported to the cytoplasm via MEX1 for respiration-dependent energy production (Niittylä et al. 2004). Maltose, maltooligosaccharides, and starch hyperaccumulate in the chloroplasts of *mex1* leaves, which also show chlorosis (Niittylä et al. 2004; Stettler et al. 2009). Electron microscopy of *mex1* leaves revealed the accumulation of abnormal chloroplasts in the cytoplasm and the occurrence of chloroplast degradation in the vacuole (Stettler et al. 2009). However, genetic evidence supporting the involvement of core ATG-dependent chlorophagy in *mex1* had not been found.

We previously identified a macroautophagy process directed toward the degradation of chloroplast components (Ishida et al. 2008; Izumi et al. 2015). This type of autophagy sequesters a portion of the chloroplast stroma and envelope into autophagosomal cargos termed Rubisco-containing bodies (RCBs), which are then transported into the vacuole (Izumi et al. 2024). RCB production is active under sugar-starvation conditions, facilitating amino acid recycling to help plants adjust to starvation (Izumi et al. 2013; Hirota et al. 2018). Our group previously explored the possible role of starch metabolism in inducing RCB-mediated macroautophagy (Izumi et al. 2010). In the leaves of Arabidopsis plants incubated in the dark, sugar-starvation phenotypes are exacerbated in starchless mutants lacking starch-producing enzymes, leading to the activation of RCB production, whereas excess starch accumulation in *sex1* or *mex1* plants suppresses RCB production, possibly because sugar starvation is never attained in these mutants (Izumi et al. 2010). These findings reveal the importance of energy availability in regulating RCB-mediated macroautophagy. However, we have not previously evaluated whether genetically altered starch metabolism influences the induction of chlorophagy for the removal of damaged chloroplasts.

In this study, we carefully assessed the incidence of chlorophagy in mutants related to maltose and starch metabolism. We obtained microscopy, transcriptome, and biochemical evidence demonstrating the induction of core ATG-dependent chlorophagy in the leaves of *mex1* mutants. We propose that *mex1* mutants could be used to help elucidate the regulatory mechanisms underlying chlorophagy.

## Results

### *Mex1* plants develop chlorotic leaves containing swollen chloroplasts

**Figure 1:**
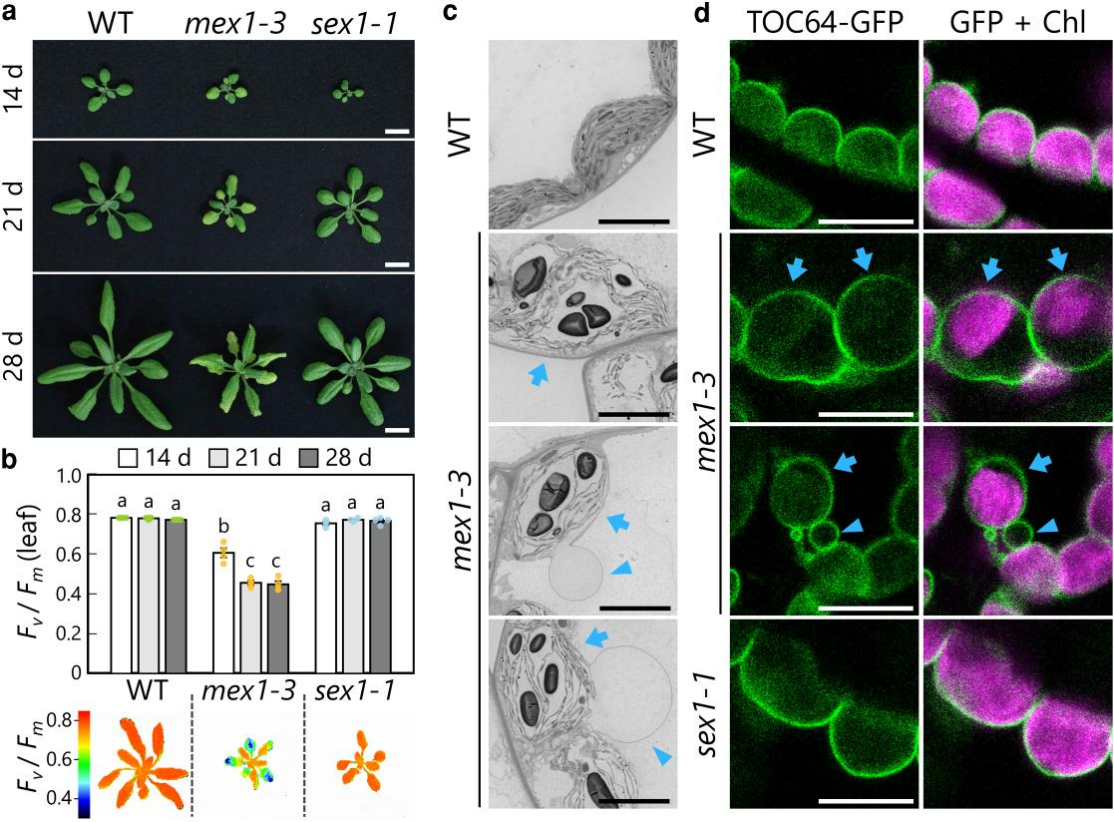
Chlorotic *mex1* leaves accumulate swollen chloroplasts. a) Representative photographs of wild-type Col-0 (WT), *mex1-3*, and *sex1-1* Arabidopsis plants grown for 14, 21, or 28 d in soil. Scale bars, 10 mm. b) Graph showing *F*_v_/*F*_m_ values measured from the second rosette leaves in 14-d-old seedlings or the third rosette leaves in 21- or 28-d-old plants per genotype as shown in a). Values are means ± standard error (SE) from 4 independent plants (*n* = 4). Dots represent individual data points; different letters denote significant differences based on Tukey's test (*P* < 0.05). Pseudo-color images indicate the *F*_v_/*F*_m_ of a whole plant shoot of 21-d-old WT, *mex1-3*, or *sex1-1* plant, as indicated by the color bar. c) Representative electron micrographs of chloroplasts from leaf mesophyll cells of 21-d-old WT and *mex1-3* plants. Arrows, swollen chloroplasts; arrowheads, globular extended chloroplast structures. Scale bars, 5 µm. d) Representative confocal microscopy images of chloroplasts from leaf mesophyll cells of 21-d-old WT, *mex1-3*, and *sex1-1* Arabidopsis plants producing the chloroplast outer envelope membrane protein TOC64-GFP. Green, TOC64-GFP; magenta, chlorophyll fluorescence (Chl). Arrows, swollen chloroplasts; arrowheads, globular extended chloroplast structures. Scale bars, 10 µm.

To explore whether chloroplast stress caused by maltose hyperaccumulation might induce chlorophagy, we evaluated *mex1* plants under our experimental conditions. When Arabidopsis plants harboring the T-DNA-insertional mutant allele *mex1-3* (SAIL_574_D11) were grown in soil, their rosette leaves turned yellow (Fig. 1a). This observation is in agreement with the decline in the maximum quantum yield of photosystem II (*F*_v_/*F*_m_), a parameter of leaf photosynthetic activity (Fig. 1b). In the *mex1-3* mutant, the *F*_v_/*F*_m_ ratio was lower in the leaves of 21- and 28-d-old plants compared to 14-d-old plants (Fig. 1b). Pseudo-color images also indicate the decline in *F*_v_/*F*_m_ value in expanded leaves relative to developing leaves in 21-d-old *mex1-3* plants (Fig. 1b, bottom). These results indicate that leaf chlorosis becomes more severe as leaves mature in *mex1-3* plants. We did not observe leaf chlorosis in wild-type (WT) or *sex1-1* plants (Fig. 1a and b); the *sex1-1* mutant served as a control due to its excess starch accumulation without concomitant maltose accumulation.

We then observed the chloroplast structures of fixed leaf samples from 21-d-old WT and *mex1-3* plants via electron microscopy. The chloroplasts of *mex1* mesophyll leaf cells had a balloon-like shape (Fig. 1c, arrows), unlike the normal structure of chloroplasts in WT leaves (Fig. 1c, top). Some *mex1* chloroplasts formed an extended globular structure (Fig. 1c, arrowheads). These observations are consistent with the results of an earlier study that identified the abnormal chloroplast morphology of *mex1-1* (Stettler et al. 2009).

To document the occurrence of these abnormal chloroplasts in living cells, we introduced a transgene encoding the chloroplast outer envelope marker TRANSLOCON AT THE OUTER MEMBRANE OF CHLOROPLASTS 64 (TOC64) fused to green fluorescent protein (TOC64-GFP) into the WT, *mex1-3*, and *sex1-1* backgrounds. TOC64-GFP fluorescence signals precisely encompassed the chlorophyll fluorescence signal in the leaves of WT and *sex1-1* plants (Fig. 1d), whereas *mex1-3* chloroplasts presented spaces without chlorophyll signal inside the TOC64-GFP-positive structures (Fig. 1d, arrows). We also observed globular structures labeled by TOC64-GFP protruding from *mex1* chloroplasts (Fig. 1d, arrowheads). Although TOC64-GFP signals visualized many stroma-filled tubules (stromules, ie, thin, tubular extensions of plastids) in epidermal cells from *mex1-3* leaves (Figure S1), such a phenomenon did not appear to occur within mesophyll chloroplasts (Figure S1).

We further defined the structure and frequency of the extended globular structures of *mex1* chloroplasts by examining plants accumulating TOC64-GFP and the chloroplast stroma marker Rubisco small subunit (RBCS) fused to monomeric red fluorescent protein (RBCS-mRFP). We detected some ring-like, globular structures of chloroplasts labeled by TOC64-GFP (9.3 in a given region) in *mex1-3* leaves, whereas such structures were absent from WT leaves (Figure S2a and b). Approximately half of the extended globules contained stromal RBCS-mRFP (Figure S2a, filled arrowheads), but the others did not (Figure S2a, open arrowheads). Since the chloroplast globules appeared to be extended into the vacuolar lumen in electron micrographs (Fig. 1c), we also observed the vacuolar membrane marker δ-TONOPLAST INTRINSIC PROTEIN (δTIP) fused to YELLOW FLUORESCENT PROTEIN (δTIP-YFP). We did not detect clear ring-like structures exhibiting δTIP-YFP signals around swollen chloroplasts in *mex1-3* leaves (Figure S2c). These observations indicate that *mex1* chloroplasts formed globular structures as part of their substructure. Overall, the electron microscopy and confocal observations revealed that mesophyll cells in chlorotic leaves of the *mex1-3* mutant accumulate abnormal chloroplasts exhibiting swelling or globularly extended envelopes.

### Chloroplasts are transported into the vacuole and then degraded in *mex1* leaves

We previously evaluated the occurrence of chlorophagy in Arabidopsis plants harboring transgenes encoding fluorescent protein markers for the chloroplast stroma or vacuolar membrane. These vacuole-delivered chloroplasts undergoing degradation via chlorophagy appear as bodies labeled by chlorophyll fluorescence but lacking fluorescence signal from marker proteins targeted to the chloroplast stroma and exhibit random movement in the vacuolar lumen (Izumi et al. 2017; Nakamura et al. 2018). To determine whether such vacuole-enclosed chloroplasts are present in the leaf mesophyll cells of *mex1-3*, we generated WT, *mex1-3*, and *sex1-1* plants harboring a transgene encoding GFP targeted to the chloroplast stroma (CT-GFP) by fusing GFP to the N-terminal transit peptide of the chloroplast protein RecA (Kohler et al. 1997).

**Figure 2:**
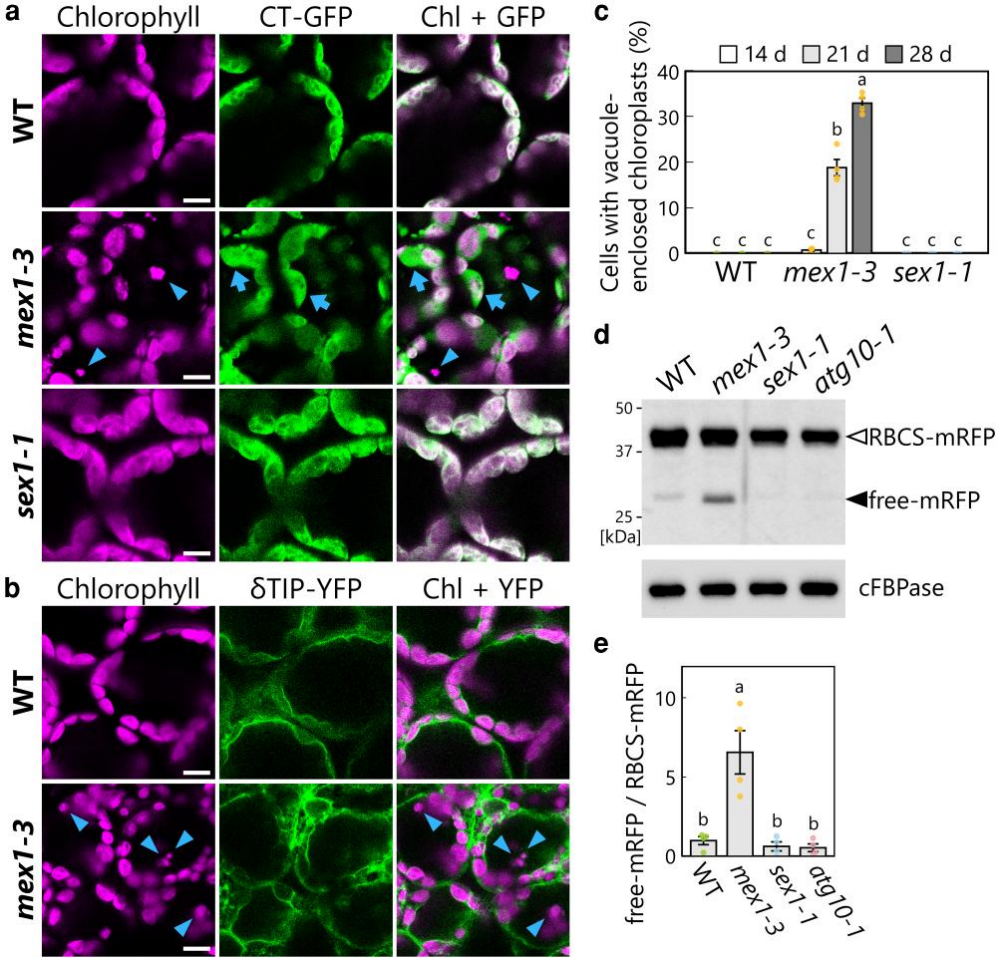
Chlorotic *mex1* leaves are sites of chloroplast degradation in vacuoles. a) Representative confocal microscopy images of leaf mesophyll cells from 21-d-old wild type Col-0 (WT), *mex1-3*, and *sex1-1* Arabidopsis plants accumulating the chloroplast stroma marker chloroplast-targeted GFP (CT-GFP). Magenta, chlorophyll fluorescence (Chl); green, CT-GFP. In the merged images, regions with overlapping GFP and chlorophyll signals appear white. Arrows, swollen chloroplasts; arrowheads, vacuole-enclosed chloroplasts. Scale bars, 10 µm. b) Representative confocal microscopy images of leaf mesophyll cells from 21-d-old WT and *mex1-3* plants harboring the tonoplast marker transgene *δTIP-YFP*. Magenta, chlorophyll fluorescence (Chl); green, δTIP-YFP. Arrowheads, vacuole-enclosed chloroplasts. Scale bars, 10 µm. c) Proportion of cells with vacuole-enclosed chloroplasts in fixed regions in the leaves of WT, *mex1-3*, and *sex1-1* plants based on the observations described in a). An example of vacuole-enclosed chloroplasts is indicated by arrowheads in a). The bars indicate means ± SE from 4 individual plants (*n* = 4). The dots represent data points from individual plants; different letters denote significant differences based on Tukey's test (*P* < 0.05). d) Immunoblot analysis of RFP and cFBPase (loading control) abundance in soluble protein extracts from the leaves of 21-d-old WT, *mex1-3*, *sex1-1*, and *atg10-1* plants harboring the *RBCS-mRFP* transgene. The open arrowhead indicates RBCS-mRFP, and the filled arrowhead indicates free mRFP derived from autophagic degradation of RBCS-mRFP. Confocal images of leaves under the same conditions are shown in Figure S2. e) Quantification of the free mRFP to RBCS-mRFP ratio from the assays described in d), normalized to that of WT plants, which was set to 1. The bars indicate means ± SE from 4 individual samples (*n* = 4). The dots represent data points from individual samples; different letters denote significant differences based on Tukey's test (*P* < 0.05).

The normally stromal CT-GFP extended beyond the region highlighted by chlorophyll fluorescence in *mex1-3* leaves (Fig. 2a, arrows), in agreement with the above observations with TOC64-GFP (Fig. 1d). Notably, we detected chlorophyll bodies located in the central areas of leaf mesophyll cells from 21-d-old and 28-d-old *mex1-3* plants (Fig. 2a, arrowheads). We confirmed the accumulation of chloroplasts in the vacuoles of *mex1-3* leaf mesophyll cells using the vacuolar membrane marker δTIP-YFP. We detected chlorophyll-labeled bodies inside the δTIP-YFP-labeled vacuolar membranes of *mex1-3* leaf mesophyll cells but not those of the WT (Fig. 2b, arrowheads).

We quantified the transport of chloroplasts to the vacuole by counting the proportion of leaf mesophyll cells containing vacuole-enclosed chloroplasts in a given region of the leaves in WT, *sex1-1*, and *mex1-3* plants harboring the *CT-GFP* transgene (Fig. 2c). Indeed, vacuole-enclosed chloroplasts accumulated in leaf mesophyll cells of *mex1-3*, but not WT or *sex1-1*. In *mex1-3* plants, we observed very few (<0.8% of observed mesophyll cells) vacuole-enclosed chloroplasts in leaf mesophyll cells from 14-d-old seedlings (Fig. 2c). By contrast, many *mex1-3* leaf mesophyll cells accumulated vacuole-enclosed chloroplasts in 21-d-old and 28-d-old plants (Fig. 2c), which showed more pronounced leaf chlorosis than 14-d-old seedlings (Fig. 1a and 1b).

To confirm the occurrence of vacuolar chloroplast degradation in *mex1* leaf cells, we examined other mutant alleles of *MEX1* (*mex1-1* and *mex1-4*; Figure S3a). The *mex1-1* allele harbors a point mutation that replaces the sequence encoding Trp-246 with a premature stop codon (Niittylä et al. 2004). The *mex1-4* allele is another T-DNA insertional mutant line isolated in this study (SALK_201638; Figure S3a). These *mex1* plants showed the same visible leaf-chlorosis phenotype and decline in *F_v_*/*F*_m_ values as *mex1-3* plants (Figure S3b and c). We grew WT plants and all 3 *mex1* alleles harboring a transgene encoding the chloroplast stroma marker RBCS-mRFP for 21 d. Chloroplasts accumulated in the vacuoles of leaf mesophyll cells from *mex1-1*, *mex1-4*, and *mex1-3* plants, but not WT plants (Figure S3d and e). These observations reveal that chloroplasts are degraded in the vacuoles of *mex1* leaf mesophyll cells.

### Biochemical evidence supports chloroplast degradation in *mex1* leaf mesophyll cells

We aimed to obtain independent evidence to corroborate the chloroplast degradation observed by microscopy in *mex1* leaf mesophyll cells. When fusion proteins consisting of an endogenous protein and a fluorescent protein, the latter being tolerant of acidic pH and lytic degradation, are transported into the vacuole or lysosomes via autophagy, the fluorescent protein is cleaved from the endogenous protein and accumulates free from its tagged protein (Mizushima et al. 2010). The accumulation of such free fluorescent protein can be detected by immunoblotting and reflects autophagic flux. RBCS-mRFP is a good indicator of the autophagic flux of chloroplast components (Ono et al. 2013; Kikuchi et al. 2020).

We therefore grew WT, *mex1-3*, *sex1-1*, and *atg10-1* (defective in a core ATG protein) plants harboring the *RBCS-mRFP* transgene for 21 d in soil and subjected them to protein analysis by immunoblotting (Fig. 2d and e). In WT leaves, the band of free mRFP was faint and the ratio of free mRFP to RBCS-mRFP was as low as that in *atg10-1* leaves, reflecting the inactive state of chlorophagy in WT leaves. We detected much more free mRFP in the soluble protein fraction from *mex1-3* leaf extracts than in extracts from the other genotypes (Fig. 2d). The ratio of free mRFP to RBCS-mRFP was 6.6 times higher in *mex1-3* leaves than WT leaves (Fig. 2e). Live confocal microscopy of leaf mesophyll cells revealed the marked accumulation of chloroplasts only in the vacuolar lumen of *mex1-3* leaves (Figure S4a, arrowheads). When we increased the brightness of the images, faint RFP signals spreading in the vacuolar lumen of *mex1-3* leaves appeared (Figure S4a, right panels). The intensity of mRFP fluorescence in the vacuolar lumen was higher in *mex1-3* than in WT, *sex1-1*, and *atg10-1* leaves (Figure S4b), which is indicative of mRFP accumulation in the vacuole. This finding is in agreement with the biochemical detection of free mRFP described above (Fig. 2d and e). These results strongly support the notion that vacuolar chloroplast degradation occurs in *mex1* leaf mesophyll cells.

### Maltose accumulation causes chloroplast degradation in *mex1* leaf mesophyll cells

The maltose hyperaccumulation phenotype in *mex1-1* was abolished by adding the *pgm-1* mutation, as the absence of the starch biosynthesis enzyme PGM blocks starch storage (Stettler et al. 2009). The rate of starch breakdown at night is lower in *sex1* leaves than the WT due to the lack of the starch-degrading enzyme GWD, resulting in a diminished maltose supply. Consequently, the hyperaccumulation of maltose in *mex1-1* plants is suppressed in the *mex1-1 sex1-3* double mutant (Stettler et al. 2009). Given that the chloroplast degradation seen in *mex1* leaves was directly linked to maltose hyperaccumulation in the chloroplasts, we reasoned that chloroplast degradation should be suppressed in *mex1 pgm* or *mex1 sex1* double mutant leaves. To test this notion, we generated the *mex1-3 pgm-1* and the *mex1-3 sex1-1* double mutant lines.

**Figure 3:**
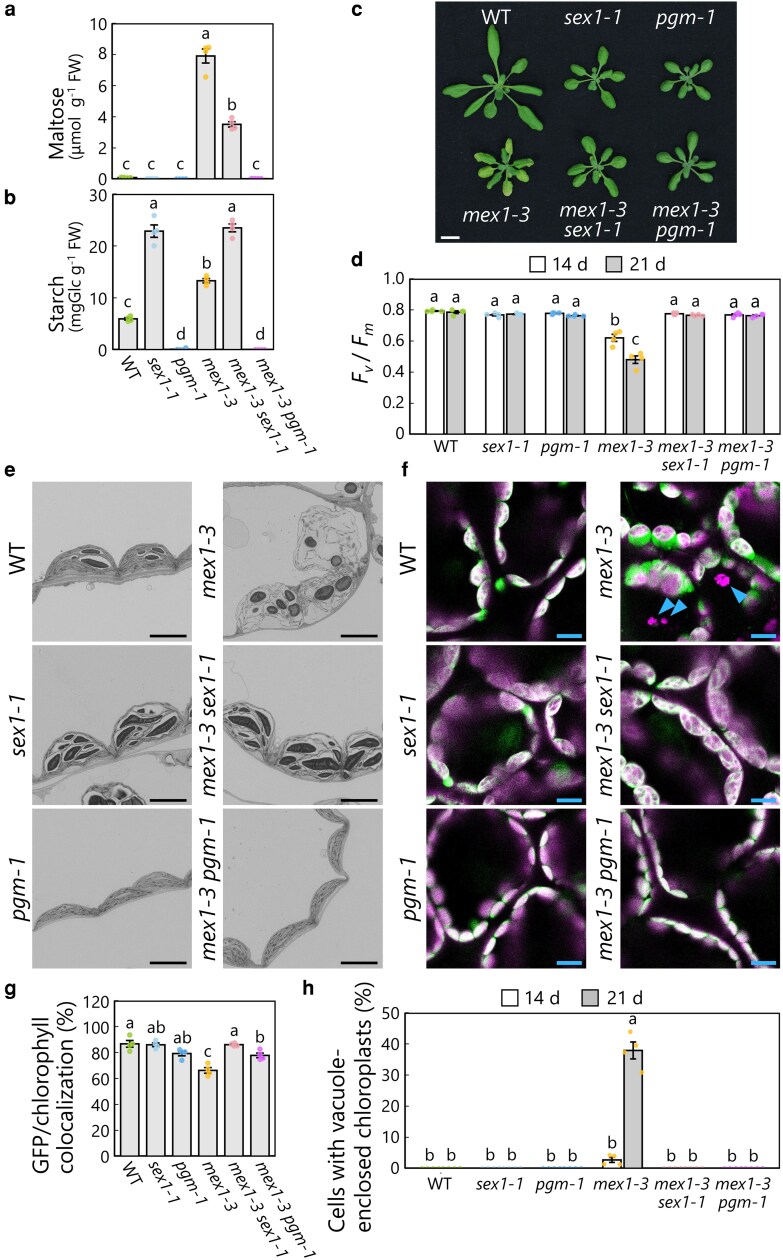
Chlorosis and vacuolar chloroplast degradation in *mex1* leaves are caused by maltose hyperaccumulation. a) Maltose contents in leaf extracts from 21-d-old wild-type Col-0 (WT), *sex1-1*, *pgm-1*, *mex1-3*, *mex1-3 sex1-1*, and *mex1-3 pgm-1* plants harvested at dawn. Glucose and sucrose contents in the same samples are shown in Figure S5. Values are means ± SE from 4 individual samples (*n* = 4). b) Starch contents in leaf extracts from the genotypes described in a) harvested in the middle of the day (7 h after dawn). Values are means ± SE from 4 individual samples (*n* = 4). c) Representative photographs of the genotypes described in a) grown in soil for 21 d. Scale bar, 10 mm. d) *F*_v_/*F*_m_ values measured from the leaves of the genotypes described in a). Values are means ± SE from 4 individual plants (*n* = 4). Second or third rosette leaves from 14-d-old or 21-d-old plants were analyzed, respectively. e) Representative electron micrographs of chloroplasts from the leaves of the genotypes described in a). Scale bars, 5 µm. Additional images of the larger areas are shown in Figure S5b. f) Representative confocal images of leaf mesophyll cells from the genotypes described in a) harboring the *CT-GFP* transgene. Green, CT-GFP; magenta, chlorophyll fluorescence (Chl). Only merged images are shown. Arrowheads, vacuole-enclosed chloroplasts. Scale bars, 10 µm. g) Percentage of GFP fluorescence volume that colocalizes with chlorophyll fluorescence in the samples shown in f). The bars indicate means ± SE from 4 individual plants (*n* = 4). h) Proportion of cells with vacuole-enclosed chloroplasts in the samples shown in f). An example of vacuole-enclosed chloroplasts is indicated by arrowheads in f). Second or third rosette leaves from 14-d-old or 21-d-old plants were analyzed, respectively. The bars indicate means ± SE from 4 individual plants (*n* = 4). In each plot, dots represent data points from individual plants or samples; different letters denote significant differences based on Tukey's test (*P* < 0.05).

We measured soluble sugar (glucose, maltose, and sucrose) and starch contents in leaf extracts from WT, *sex1-1*, *pgm-1*, and *mex1-3* single mutant plants and *mex1-3 pgm-1* and *mex1-3 sex1-1* double mutant plants (Fig. 3a and b; Figure S5a). Maltose content was 77.8 times higher in *mex1-3* leaves than in WT leaves (Fig. 3a). Similar drastic increases were not detected in glucose or sucrose contents (Figure S5a). Maltose accumulation did not occur in *mex1-3 pgm-1*, WT, *sex1-1*, or *pgm-1* leaves (Fig. 3a). The maltose content in *mex1-3 sex1-1* leaves decreased to approximately 44.5% that of the *mex1-3* single mutant. Starch accumulated to similarly high levels in *sex1-1* and *mex1-3 sex1-1* leaves (Fig. 3b). Starch did not accumulate in the leaves of *pgm-1* or *mex1-3 pgm-1* plants, reflecting the lack of starch biosynthesis due to the loss of PGM function. These results confirm the notion that maltose hyperaccumulation due to the mutation of *MEX1* is fully or partially suppressed by the addition of mutation in *PGM* or *SEX1*, respectively.

The mitigation of maltose accumulation influenced the visible phenotypes of the double mutant plants. Specifically, 21-d-old *mex1-3 sex1-1* and *mex1-3 pgm-1* plants did not show the leaf-chlorosis phenotype characteristic of the *mex1-3* single mutant (Fig. 3c). The *F*_v_/*F*_m_ values measured from the leaves of *mex1-3 sex1-1* and *mex1-3 pgm-1* plants were as high as those in WT leaves when the plants were grown for 14 d or 21 d, in sharp contrast to the lower *F*_v_/*F*_m_ values of *mex1-3* leaves (Fig. 3d). Electron microscopy of chloroplast ultrastructure indicated that the *pgm* or *sex1* mutation mitigated the membrane stress of *mex1* chloroplasts (Fig. 3e; Figure S5b): *pgm-1* and *pgm-1 mex1-3* mesophyll cells contained elliptic and starch granule-less chloroplasts, and *sex1-1* and *sex1-1 mex1-3* chloroplasts accumulated large starch granules without swelling and globular extensions, which were seen in *mex1-3* chloroplasts. We introduced the *CT-GFP* transgene into these mutant backgrounds to observe chloroplast behavior in their living leaves. The chloroplast swelling phenotype seen in *mex1-3* leaf mesophyll cells was abolished by the presence of the *pgm-1* or *sex1-1* mutation (Fig. 3f). To quantify these morphological changes, we calculated the proportion of colocalization between the stromal CT-GFP signal and the chlorophyll fluorescence signal (Fig. 3g). This colocalization ratio will drop when chloroplast swelling occurs, as the swollen regions are only marked by CT-GFP but not by the chlorophyll signal. Indeed, the colocalization ratio was lower in *mex1-3* than in WT and *sex1-1* leaves (Fig. 3g) but returned to levels close to those seen in WT upon the addition of the *sex1-1* or *pgm-1* mutation. Importantly, we did not detect vacuole-enclosed chloroplasts in *mex1-3 sex1-1* or *mex1-3 pgm-1* leaf mesophyll cells, unlike in *mex1-3* cells (Fig. 3f, arrowheads, and 3h). These results indicate that the lower maltose accumulation in *mex1-3 pgm-1* or *mex1-3 sex1-1* leaves mitigates chloroplast swelling and subsequently suppresses chloroplast degradation.

### Chloroplast degradation in *mex1* leaf mesophyll cells requires the core ATG machinery

**Figure 4:**
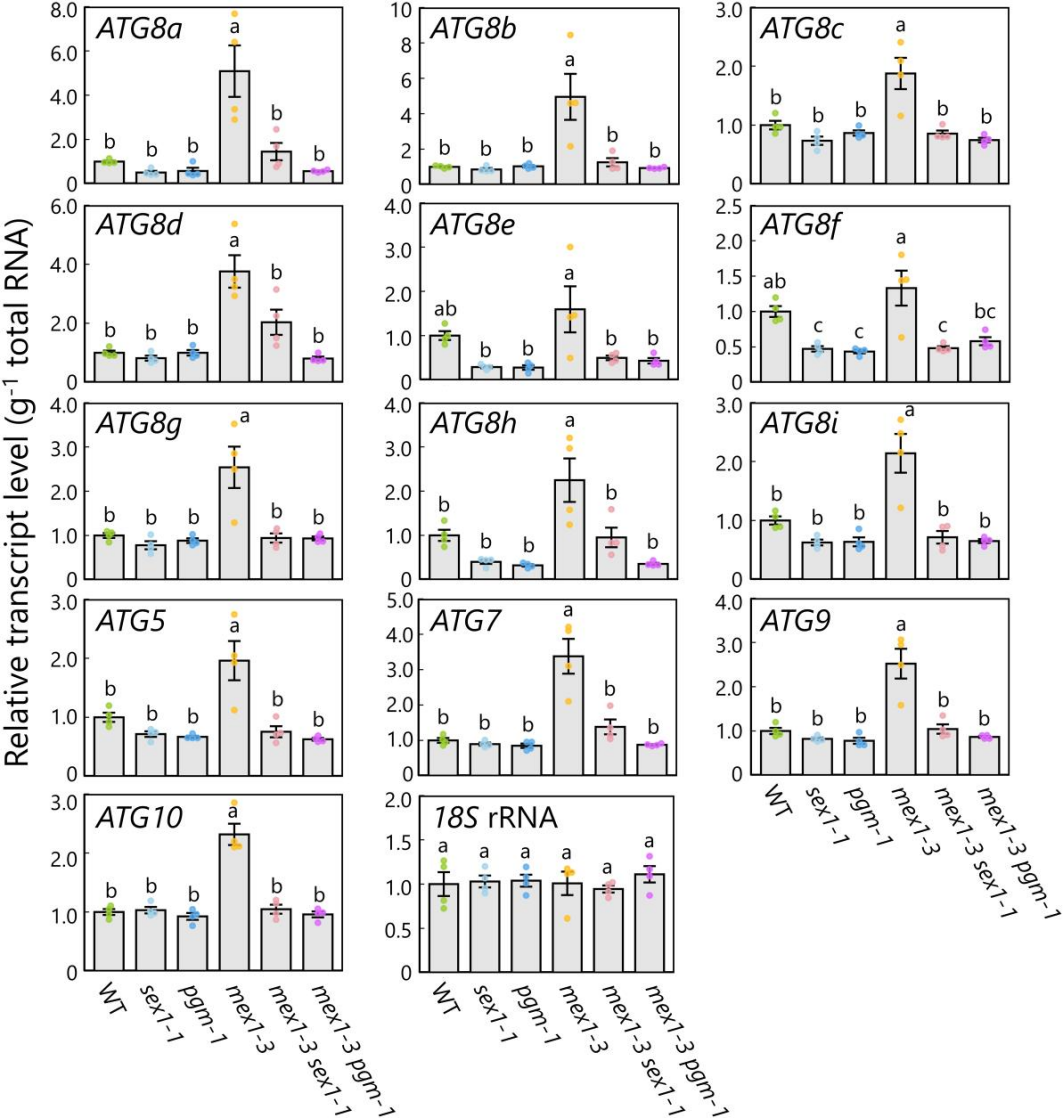
The expression levels of autophagy-related genes are higher in the *mex1-3* mutant, which accumulates maltose. Relative transcript levels of the autophagy-related genes *ATG8a–i*, *ATG5*, *ATG7*, *ATG9*, and *ATG10* in the leaves of 21-d-old wild-type Col-0 (WT), *sex1-1*, *pgm-1*, *mex1-3*, *mex1-3 sex1-1*, and *mex1-3 pgm-1* plants relative to the values in WT leaves, which were set to 1. The level of *18S* rRNA was measured as an internal control. The bars indicate mean transcript levels ± SE from 4 individual samples (*n* = 4). In each plot, dots represent data points from individual samples; different letters denote significant differences based on Tukey's test (*P* < 0.05).

We turned our attention to the involvement of the core autophagy machinery in the chloroplast degradation program induced by maltose hyperaccumulation. We measured the transcript levels of core *ATG* genes in the leaves of 21-d-old plants. Among the *ATG* genes examined, the relative abundances of *ATG8a–d*, *ATG8g–i*, *ATG5*, *ATG7*, *ATG9*, and *ATG10* transcripts were higher in the leaves of *mex1-3* plants than the WT (Fig. 4). This upregulation was counteracted in *mex1-3 pgm-1* and *mex1-3 sex1-1* plants, whose transcript abundances largely returned to WT levels (Fig. 4). We also examined the transcript levels of *ATG* genes in the 3 *mex1* mutant alleles and found that *ATG8a*, *ATG8b*, *ATG8d*, *ATG8e*, *ATG5*, *ATG7*, *ATG9*, and *ATG10* were commonly upregulated in all 3 alleles compared to WT (Figure S6). These changes in transcript levels indicate that maltose hyperaccumulation in chloroplasts and the leaf-chlorosis phenotype are associated with elevated transcript levels of a subset of *ATG* genes.

**Figure 5:**
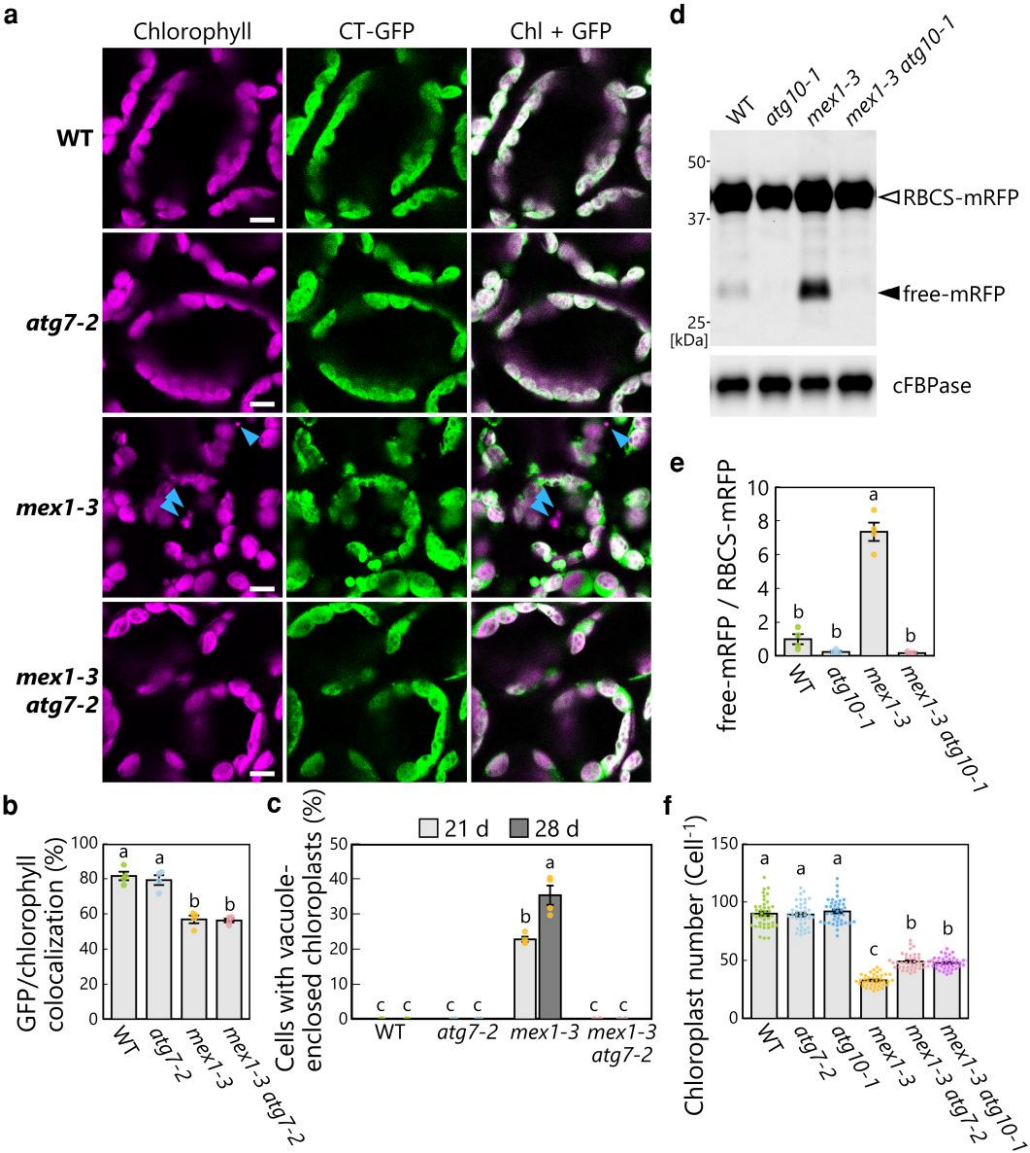
Vacuolar chloroplast degradation in the leaves of *mex1* plants requires the core autophagy machinery. a) Representative confocal images of leaf mesophyll cells from 21-d-old wild-type Col-0 (WT), *atg7-2*, *mex1-3*, and *mex1-3 atg7-2* plants harboring the *CT-GFP* transgene. Magenta, chlorophyll fluorescence (Chl); green, CT-GFP. Arrowheads, vacuole-enclosed chloroplasts. Scale bars, 10 µm. b) Percentages of GFP fluorescence volume that colocalize with chlorophyll fluorescence based on the observations described in a). The bars indicate means ± SE from 4 individual plants (*n* = 4). c) Proportion of cells with vacuole-enclosed chloroplasts based on the observations described in a). An example of vacuole-enclosed chloroplasts is indicated by arrowheads in a). The bars indicate means ± SE from 4 individual plants (*n* = 4). d) Immunoblot analysis of RFP and cFBPase (loading control) abundance in soluble protein extracts from the leaves of 21-d-old WT, *atg10-1*, *mex1-3*, and *mex1-3 atg10-1* plants harboring the *RBCS-mRFP* transgene. Open arrowhead, RBCS-mRFP; filled arrowhead, free mRFP derived from autophagic degradation of RBCS-mRFP. Confocal images of leaves under the same conditions are shown in Figure S7. e) Quantification of the free mRFP to RBCS-mRFP ratio from the assays described in d) relative to that in WT plants, which was set to 1. Values are means ± SE from 4 individual samples (*n* = 4). (f) Chloroplast number per mesophyll cell measured in chemically fixed leaves from 21-d-old plants of the indicated genotypes. Representative images of fixed cells are shown in Figure S8. The bars indicate means ± SE from 40 chloroplasts (*n* = 40) from 4 individual plants. In each plot, dots represent data points from individual plants, samples, or chloroplasts; different letters denote significant differences based on Tukey's test (*P* < 0.05).

To directly examine the involvement of the core ATG machinery in chloroplast degradation in *mex1*, we generated double mutants of *mex1* and genes encoding core ATG components. First, we obtained the *mex1-3 atg7-2* double mutant line harboring the *CT-GFP* transgene as a stromal marker. When WT, *atg7-2*, *mex1-3*, and *mex1-3 atg7-2* plants were grown for 21 d, chloroplast swelling occurred in *mex1-3* and *mex1-3 atg7-2* leaf mesophyll cells (Fig. 5a), which was reflected by the lower colocalization ratio between stromal CT-GFP signal and chlorophyll signal in *mex1-3* and *mex1-3 atg7-2* compared to WT and *atg7-2* (Fig. 5b). However, vacuole-enclosed chloroplasts accumulated only in the leaves of 21-d-old and 28-d-old *mex1-3* plants (Fig. 5c). These results indicate that the loss of ATG7 function in *mex1 atg7* leaves suppresses the chloroplast degradation seen in *mex1* leaves without affecting chloroplast swelling.

We independently performed an RBCS-mRFP cleavage assay in WT, *atg10-1*, *mex1-3*, and *mex1-3 atg10-1* plants carrying the *RBCS-mRFP* transgene. The accumulation of free mRFP in *mex1-3* leaves was clearly abolished in *mex1-3 atg10-1* leaves (Fig. 5d and e). Confocal microscopy of leaf mesophyll cells revealed the accumulation of chloroplast fragments (as evidenced by chlorophyll fluorescence) and the spread of RFP signal in the vacuolar lumen only in *mex1-3* leaf mesophyll cells (Figure S7a and b). We further assessed the impact of chlorophagy on the chloroplast population by measuring chloroplast number in chemically fixed mesophyll cells (Fig. 5f; Figure S8). The *mex1-3* mutation caused a decrease in chloroplast number, likely due to the inhibited chloroplast division and cell expansion during leaf development. Notably, chloroplast number decreased in *mex1-3* cells relative to *mex1-3 atg7-2* and *mex1-3 atg10-1*, which is in agreement with the finding that chlorophagy was induced in *mex1-3* leaves and suppressed by the *atg7-2* or *atg10-1* mutation. These results support the notion that core ATG-dependent chlorophagy is induced by maltose hyperaccumulation in chloroplasts.

Macroautophagy of chloroplast stroma components with RCB production can also contribute to the accumulation of cleaved mRFP from RBCS-mRFP in the vacuole, especially in leaves during incubation in the dark accompanied by sugar starvation (Izumi et al. 2024). We thus tested the activity of this type of autophagy by incubating WT, *mex1-3*, and *sex1-1* plants harboring the *CT-GFP* transgene in the dark with concanamycin A (conA), an inhibitor of vacuolar H^+^-ATPase. Confocal microscopy of WT leaf mesophyll cells following dark treatment detected RCBs approximately 1 µm in diameter as bright puncta labeled with CT-GFP (Figure S9a, arrowheads). Fewer RCBs accumulated in *mex1-3* and *sex1-1* leaf mesophyll cells than in WT (Figure S9b). These results are similar to previous findings (Izumi et al. 2010). These results suggest that the accumulation of free mRFP derived from RBCS-mRFP cleavage in *mex1* leaves is largely associated with active chlorophagy, but not with RCB-mediated macroautophagy.

**Figure 6:**
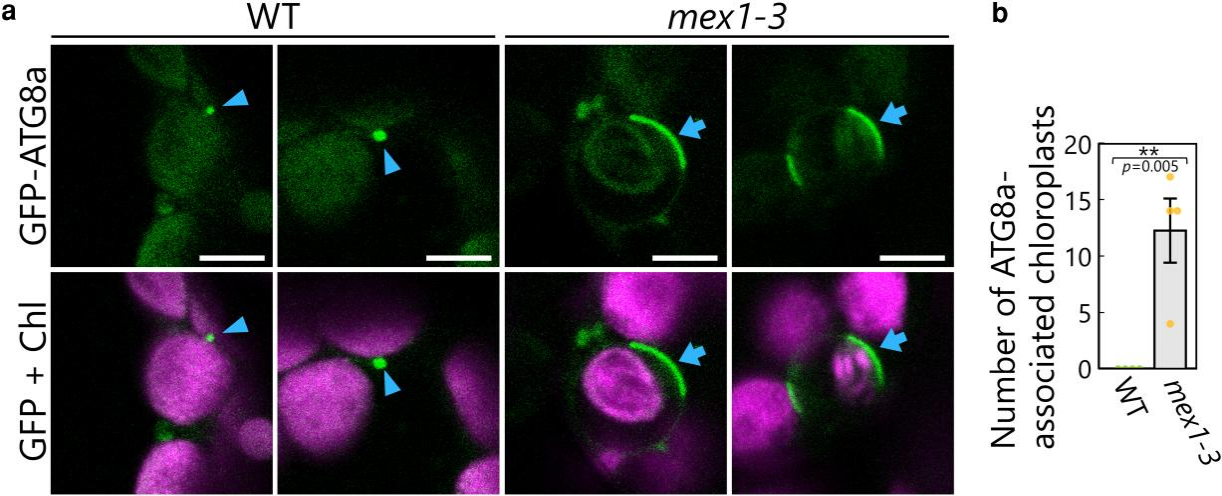
ATG8-labeled flattened structures accumulate on swollen chloroplasts in *mex1* leaves. a) Representative confocal images of leaf mesophyll cells from 21-d-old wild-type Col-0 (WT) and *mex1-3* plants accumulating the autophagosomal membrane marker GFP-ATG8a. Green, GFP-ATG8a; magenta, chlorophyll fluorescence (Chl). Arrowheads, GFP-ATG8a-labeled cytoplasmic puncta; arrows, GFP-ATG8a-associated chloroplasts. Scale bars, 5 µm. b) Number of ATG8-associated chloroplasts in a given region based on the observations described in a). The bars indicate means ± SE from 4 individual plants (*n* = 4). Dots represent data points from individual plants; asterisks denote significant differences based on *t*-test (**P < 0.01).

The accumulation of ATG8-labeled structures on the chloroplast surface is also a characteristic of core ATG-dependent chlorophagy (Nakamura et al. 2018). To examine whether this phenomenon also occurs in *mex1* plants, we introduced a construct expressing GFP-ATG8a into the WT and *mex1-3* backgrounds. Although we detected GFP-ATG8a signal as cytoplasmic puncta in WT leaf mesophyll cells (Fig. 6a, arrowheads), the GFP signal formed large, flattened structures in contact with the surfaces of chloroplasts in *mex1-3* (Fig. 6a, arrows). The observation of *mex1-3* leaves producing GFP-ATG8a and TOC64-mRFP more clearly revealed the accumulation of GFP-ATG8a signals on the surfaces of swelling regions of *mex1* chloroplasts (Figure S10a). We also detected chloroplasts undergoing digestion in the vacuole (Figure S10b, filled arrowheads), along with cytoplasmic chloroplasts surrounded by GFP-ATG8a signals (Figure S10b, open arrowheads) in *mex1-3* leaves. Chloroplasts labeled by large GFP-ATG8a structures were not observed in WT plants (Fig. 6b). Taken together, we conclude that chlorophagy that requires the core autophagy machinery occurs in *mex1-3* leaf mesophyll cells.

NEIGHBOR OF BRCA1 GENE 1 (NBR1) is an evolutionarily conserved autophagy adaptor protein that connects ubiquitinated substrates to ATG8 for autophagic degradation. However, a recent study revealed that NBR1 mediates the degradation of damaged chloroplasts independently of the core ATG machinery (Lee et al. 2023). We thus created a construct producing NBR1-GFP and observed its localization in WT and *mex1-3* leaves (Figure S11). NBR1-GFP signals appeared as cytoplasmic puncta in WT and *mex1-3* leaves (Figure S11a, arrows). Although we did not detect the accumulation of NBR1-GFP in flattened structures around swollen chloroplasts in *mex1-3* leaves, a few chloroplasts accumulated dot-like NBR1-GFP signals in their interiors (Figure S11a, arrowheads). However, the frequency of such chloroplasts (1.8 in a given region; Figure S11b) was much lower than that of GFP-ATG8a-associated chloroplasts (11.5 in a given region; Fig. 6b). These results indicate that core ATG machinery-dependent chlorophagy primarily mediates the degradation of chloroplasts in *mex1* leaves.

### Autophagy deficiency alleviates the leaf-chlorosis phenotype caused by maltose hyperaccumulation

**Figure 7:**
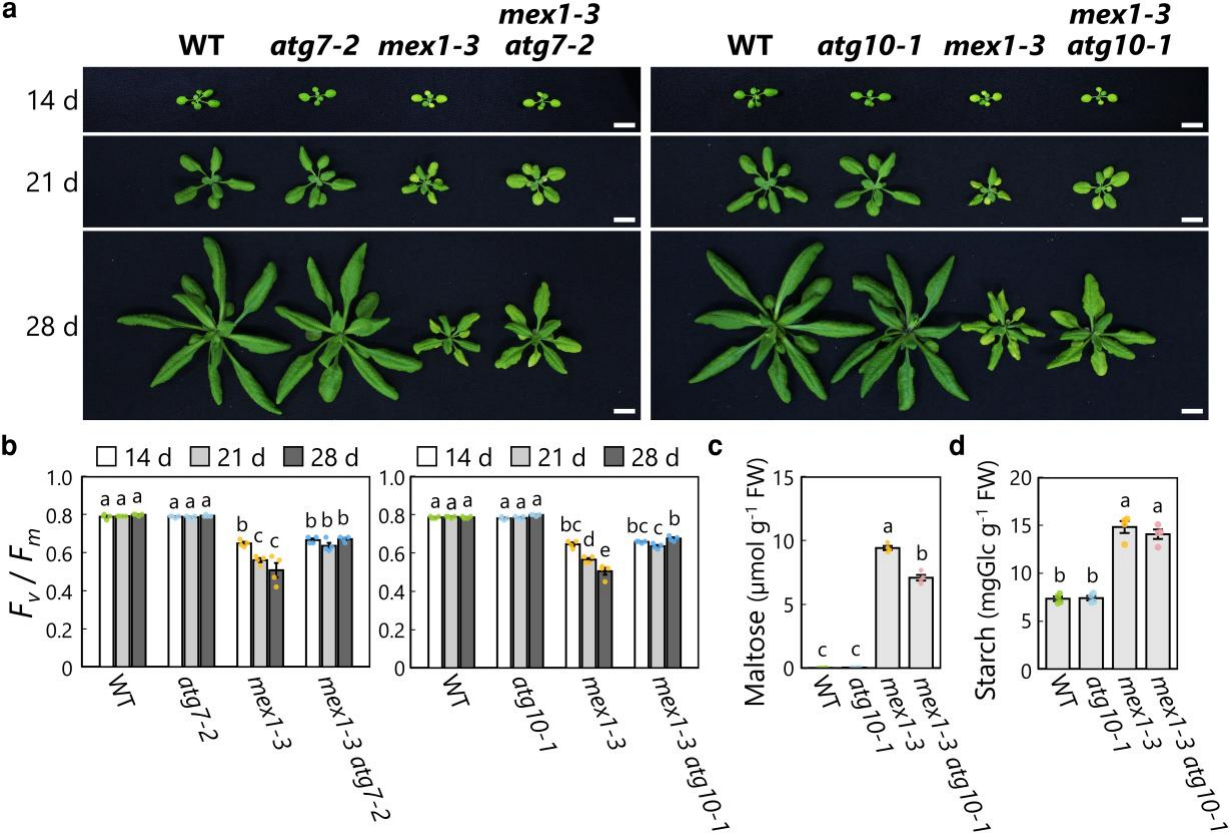
Autophagy deficiency alleviates the leaf-chlorosis phenotype of maturing *mex1* plants. a) Representative photographs of wild-type Col-0 (WT), *atg7-2*, *mex1-3*, and *mex1-3 atg7-2* seedlings or plants (left panels), or WT, *atg10-1*, *mex1-3*, and *mex1-3 atg10-1* seedlings or plants (right panels), grown for 14, 21, or 28 d in soil. Scale bars, 10 mm. b) *F*_v_/*F*_m_ values measured from the leaves of the genotypes described in a). Values are means ± SE from 4 individual plants (*n* = 4). c) Maltose contents in leaf extracts from 21-d-old WT, *atg10-1*, *mex1-3*, and *mex1-3 atg10-1* plants harvested at dawn. Values are means ± SE from 4 individual samples (*n* = 4). d) Starch contents in leaf extracts from the genotypes described in c) harvested in the middle of the day (7 h after dawn). Values are means ± SE from 4 individual samples (*n* = 4). In each plot, dots represent data points from individual plants or samples; different letters denote significant differences based on Tukey's test (*P* < 0.05).

Chlorophagy in *mex1* was active in the leaves of 21-d-old and 28-d-old plants, which exhibited greater leaf chlorosis than the leaves of 14-d-old seedlings (Figs. 1 and 5). We therefore hypothesized that chlorophagy exacerbates leaf chlorosis in maturing *mex1* leaves. To investigate this possibility, we grew 2 sets of plants and carefully compared their growth: one set comprised WT, *atg7-2*, *mex1-3*, and *mex1-3 atg7-2* plants and the other set consisted of WT, *mex1-3*, *atg10-1*, and *mex1-3 atg10-1* plants (Fig. 7a). Notably, *mex1-3 atg7-2* and *mex1-3 atg10-1* plants produced larger rosettes than *mex1-3* single mutant plants at 21 d or 28 d after sowing, whereas seedlings of the same double mutants were the same size as *mex1-3* single mutant seedlings at 14 d after sowing (Fig. 7a). Similarly, the *F*_v_/*F*_m_ values were higher in the leaves of double mutant vs. *mex1-3* single mutant plants at 21 or 28 d old, whereas the *F*_v_/*F*_m_ values were similar in leaves from 14-d-old *mex1-3*, *mex1-3 atg7-2*, and *mex1-3 atg10-1* seedlings (Fig. 7b). Therefore, the exacerbation of leaf chlorosis in maturing *mex1* leaves from 21-d-old or 28-d-old plants was suppressed by the additional loss of ATG7 or ATG10. We obtained similar results for other double mutant combinations with different *mex1* alleles (*mex1-1* and *mex1-4*) and *atg7-2* (Figure S12). These results indicate that the deficiency of autophagy mitigates the aggravated chlorosis seen in maturing *mex1* leaves, but does not completely alleviate the leaf-chlorosis phenotype in young *mex1* seedlings.

We measured maltose and starch contents in WT, *mex1-3*, *atg10-1*, and *mex1-3 atg10-1* leaves at 21 d after sowing. Although the maltose content of *mex1-3 atg10-1* leaves dropped to 75.3% compared to *mex1-3* leaves, it was still 149 times higher than in WT leaves (Fig. 7c). Starch content was twice as high in *mex1-3* and *mex1-3 atg10-1* leaves compared to WT and *atg7-2* leaves (Fig. 7d). Confocal microscopy observations indicated that chloroplast swelling occurs in *mex1-3 atg7-2* and *mex1-3 atg10-1* (Fig. 5; Figure S7). These results suggest that the *atg* mutations did not completely mitigate the maltose hyperaccumulation stress within chloroplasts caused by the *mex1-3* mutation.

We directly compared the phenotypic rescue observed in *mex1-3 atg10-1* to that in *mex1-3 sex1-1*, which showed no pale-green leaves from the young seedling stage (Fig. 3a to d). We grew WT, *atg10-1*, *sex1-1*, *mex1-3*, *mex1-3 atg10-1*, and *mex1-3 sex1-1* plants side by side (Figure S13). During early growth (14 d after sowing), leaf chlorosis and the lower *F*_v_/*F*_m_ values in *mex1* plants were completely suppressed by the addition of the *sex1-1* mutation, returning to WT levels, but were not suppressed by the addition of the *atg10-1* mutation (Figure S13a and b). By contrast, the *atg10-1* mutation only partially mitigated the leaf chlorosis in *mex1-3* at 21 d and 28 d after sowing. Therefore, we conclude that the partial phenotypic rescue of *mex1* by the additional *atg* mutation engages a strategy distinct from the rescue observed in *mex1 sex1* double mutant plants, highlighting the possibility that active chlorophagy due to maltose hyperaccumulation exacerbates leaf chlorosis in maturing *mex1* plants.

### Maltose hyperaccumulation and high-light damage stimulate common transcriptomic responses

The above experiments suggested that maltose-hyperaccumulation stress in *mex1* leaves leads to overactive chlorophagy, resulting in the aggravation of leaf chlorosis. Previous studies indicated that chloroplast swelling and chlorophagy are also activated in leaves exposed to strong light (Izumi et al. 2017; Nakamura et al. 2018). We hypothesized that these 2 different conditions (maltose hyperaccumulation in chloroplasts and strong light damage) may share some responses, including key signals in chlorophagy induction. Therefore, to compare the transcriptional responses caused by *mex1* mutation and strong light damage, we performed transcriptome deep sequencing (RNA-seq) of Arabidopsis leaf samples from 2 experimental settings: one set consisted of leaves collected from 21-d-old WT, *mex1-3*, and *sex1-1* plants, while the other consisted of leaves from WT plants 1 d after exposure to transient high light for 2 h (HL; 2,000 µmol m^−2^ s^−1^) and control WT plants, as HL treatment.

**Figure 8:**
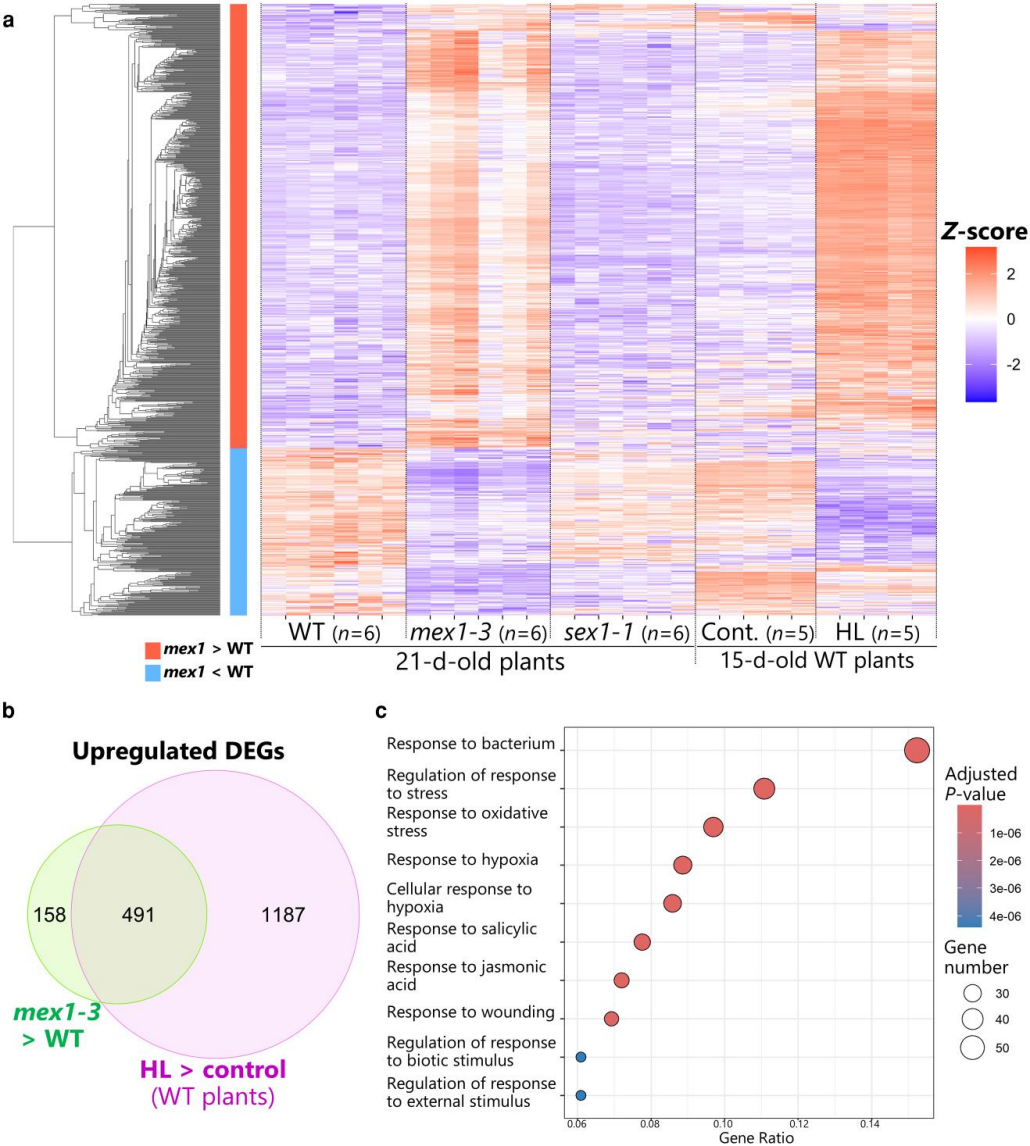
Maltose accumulation stress in the *mex1* mutant and photodamage due to high-light exposure share similar transcriptome profiles. a) Heatmap representation of gene expression levels in the leaves of 21-d-old wild-type Col-0 (WT) and *mex1-3* or *sex1-1* plants and the leaves of 15-d-old WT plants 1 d after 2 h of high-light treatment (HL) or from untreated plants (Cont). Eight hundred ninety-three differentially expressed genes (DEGs) in *mex1-3* relative to WT were ordered by hierarchical clustering, and their normalized transcript levels are expressed as *Z*-scores. Each row indicates the *Z*-score of a DEG, as indicated by the color bar to the right of the heatmap. Red and blue boxes to the left of the heatmap indicate the area of DEGs that were upregulated (red) or downregulated (blue) in *mex1-3* relative to WT. Each column indicates the result from one sample. RNA-seq data are shown from 5 or 6 individual samples (*n* = 5 or 6). b) Venn diagram showing the extent of overlap between the upregulated DEGs in *mex1-3* relative to WT and following HL treatment. c) Gene Ontology (GO) enrichment analysis of shared upregulated DEGs identified in *mex1-3* and HL-exposed leaves. Simplified outputs of enriched GO (biological process) terms are shown, after applying the “Simplify” function in clusterProfiler. All enriched GO terms are listed in Table S5. Circle size indicates the number of genes enriched in each GO term. Circle colors indicate the adjusted *P*-values, as shown by the color bar.

RNA-seq of WT and *mex1-3* leaves identified 893 differentially expressed genes (DEGs) with an absolute log_2_(fold change) ≥ 1 and adjusted *P* < 0.01. Of these, 649 genes were upregulated and 244 genes were downregulated in *mex1-3* leaves compared to WT leaves (Tables S1 and S2). We calculated the *Z*-scores for all DEGs across all 5 sets of samples, performed hierarchical clustering, and represented their changes in transcript levels as a heatmap (Fig. 8a). Most genes that were upregulated in *mex1-3* vs. WT leaves were not upregulated in *sex1-1* leaves, indicating that the transcriptomic responses seen in *mex1-3* leaves are extensively associated with the maltose-excess and leaf-chlorosis phenotypes, but not with the starch-excess phenotype. Notably, many DEGs that were upregulated in *mex1-3* vs. WT leaves were also upregulated upon HL treatment (Fig. 8a). A comparison of DEGs in *mex1-3* vs. WT leaves to those in HL-exposed WT vs. untreated WT leaves identified 491 DEGs (75.7% of upregulated DEGs in *mex1-3* vs. WT leaves) that are commonly upregulated by the *mex1* mutation and HL damage (Fig. 8b; Tables S1 and S3). By contrast, 119 DEGs were commonly downregulated by the *mex1* mutation and HL damage (Figure S14a; Tables S2 and S4). We conclude that maltose hyperaccumulation stress and HL damage stress share some transcriptional responses.

Commonly upregulated DEGs by the *mex1* mutation and HL treatment included numerous transcription factor genes, such as *WRKY8*, *WRKY15*, *WRKY18*, *WRKY25*, *WRKY30*, *WRKY38*, *WRKY46*, *WRKY47*, *WRKY50*, *WRKY51*, *WRKY53*, *WRKY59*, *WRKY60*, *WRKY62*, and *WRKY75*; as well as *NAC003*, *NAC016*, *NAC036*, *NAC042*, *NAC046*, *NAC087*, and *NAC090* (Table S1). We validated the upregulation of a selected set of these transcription factor genes by reverse-transcription quantitative PCR (RT-qPCR) (*WRKY18* and *WRKY62*, and *NAC016* and *NAC042*; Figure S15). Such differences in expression suggest that maltose-excess stress and HL damage induce similar transcriptome reprogramming.

We subjected the common DEGs due to the *mex1* mutation and HL treatment to Gene Ontology (GO) enrichment analysis to explore the types of responses that were shared (Fig. 8c; Figure S14b; Tables S5 and S6). The commonly upregulated DEGs were enriched in genes related to responses to biotic stress, including bacterial infection, oxidative stress, and salicylic acid (Fig. 8c). We also observed an enrichment for GO terms related to response to wounding and jasmonic acid, the latter being the major signal from wounding stress (Fig. 8c). Therefore, maltose hyperaccumulation and HL treatment both appear to activate responses to external stimuli such as biotic stress or wounding. Genes related to photosynthesis were enriched in the commonly downregulated genes (Figure S14b), which might reflect the growth retardation phenotype due to maltose-excess stress or HL-related damage. GO enrichment analysis revealed that distinct conditions (maltose hyperaccumulation and HL damage) share some transcriptomic responses, including responses to biotic stress or wounding.

Finally, we investigated the possibility that HL damage affects starch metabolism and causes maltose hyperaccumulation, resulting in the production of swollen chloroplasts and their degradation by chlorophagy. At 1 d after HL treatment, we detected the production of swollen chloroplasts in plants accumulating TOC64-GFP and RBCS-mRFP (Figure S16a). We examined the transcriptional changes of the genes involved in starch metabolism in our RNA-seq data (Figure S16b and c). Of the 14 genes examined, only 2 (*GBSS1*, encoding granule bound starch synthase 1, and *BAM1*, encoding β-amylase 1) were upregulated by HL. Therefore, the stimulation of the starch degradation pathway, which releases maltose, was not detected at the transcriptional level. We measured changes in carbohydrate contents in response to HL stress (Figure S16d and e). No clear changes in starch contents were detected. Although maltose contents increased in HL-treated leaves relative to untreated control leaves, the extent of increase was only 1.6-fold (Figure S16e). The extent of increase in both sucrose and glucose contents was comparable to that of maltose. Therefore, HL stress did not cause a specific increase in maltose contents, which was observed in *mex1-3* leaves (Fig. 3a; Figure S5a). These results support the notion that the factor inducing chloroplast swelling under HL treatment is different from that in *mex1* leaves; however, the production of swollen chloroplasts, a common phenotype under both conditions, generates signals that are directly linked to the induction of chlorophagy.

## Discussion

Chlorophagy is a type of microautophagy that requires the core autophagy machinery and eliminates damaged chloroplasts resulting from strong light exposure (Izumi et al. 2017; Nakamura et al. 2018). We reasoned that identifying additional stress conditions that can induce chlorophagy would advance our understanding of how chlorophagy is activated. We focused on chloroplast degradation that was previously reported in mutants of *MEX1* (Stettler et al. 2009). Monitoring chlorophagy through confocal microscopy of fluorescent protein markers and biochemical detection of autophagy flux revealed that maltose hyperaccumulation in chloroplasts triggers the vacuolar transport of chloroplasts in *mex1* leaf mesophyll cells. This phenomenon was suppressed by the additional loss of core ATG proteins. ATG8-containing structures accumulated on the surfaces of *mex1* chloroplasts accumulating excess maltose. Taken together, our results reveal that core ATG-dependent chlorophagy mediates chloroplast degradation in *mex1* leaf mesophyll cells.

### How does maltose hyperaccumulation induce chlorophagy?

When Arabidopsis plants are exposed to transient HL treatment, swollen chloroplasts appear and are in contact with flattened structures containing ATG8, resulting in their removal into the vacuole (Nakamura et al. 2018). Maltose-excess stress also produced swollen chloroplasts (Fig. 1), which were associated with GFP-ATG8a-labeled structures (Fig. 6). Therefore, the current findings strengthen the idea that chloroplast swelling is a key signal that is recognized as a selective target for chlorophagy. Since maltose is a soluble sugar, its accumulation to extremely high levels in the chloroplast stroma may collapse the osmotic balance across the chloroplast envelopes, causing an influx of water into the organelle and thus chloroplast swelling. In WT leaves exposed to HL damage, a drastic, specific increase in maltose content did not occur (Figure S16e). Therefore, we propose that different inputs produce osmotically affected, swollen chloroplasts in response to the *mex1* mutation vs. HL stress. However, the swollen chloroplasts under each condition generate common signals that would allow the cytoplasmic autophagy machinery to sense these chloroplasts as a target and upregulate the transcript levels of chlorophagy-related genes in the nuclear genome. Additionally, the *mex1* mutation suppressed the macroautophagic degradation of portions of chloroplasts in leaves incubated in the dark (Figure S9) since starch hyperaccumulation prevents the exhaustion of sugars (Izumi et al. 2010). Chlorophagy to remove swollen chloroplasts and macroautophagy in response to sugar starvation are likely controlled by distinct mechanisms.

Ubiquitination is a well-known tag for organelles or protein aggregates that must be removed by autophagy. Ubiquitination-triggered selective autophagy is facilitated by so-called autophagy adaptors that connect autophagosomal membrane–anchored ATG8 with the polyubiquitin chains on their target components. NBR1 is an evolutionarily conserved autophagy adaptor protein that participates in the degradation of chloroplast proteins (Lee et al. 2023; Wan et al. 2023). We found that the NBR1-GFP accumulation pattern was clearly different from that of GFP-ATG8a in *mex1* leaves (Figure S11). This result agrees with the previous finding that NBR1 degrades damaged chloroplasts caused by strong light exposure via a mechanism independent of the canonical autophagy machinery (Lee et al. 2023). However, the frequency of GFP-ATG8a-decorated chloroplasts was much higher than that of chloroplasts accumulating NBR1-GFP signals (Fig. 6b; Figure S11b). Furthermore, the current study offers multiple lines of evidence indicating that the core autophagy machinery drives chlorophagy in *mex1* leaf mesophyll cells. Therefore, ATG-dependent chlorophagy is likely a crucial contributor to the degradation of maltose-excess chloroplasts.

RT-qPCR revealed that some *ATG* genes are upregulated in *mex1* leaves (Fig. 4; Figure S6). Maltose hyperaccumulation and subsequent leaf chlorosis might therefore also stimulate autophagy at the transcriptional level. However, a previous study investigating transcript levels in WT and *mex1-1* plants via microarrays did not detect the upregulation of *ATG* genes in *mex1-1* leaves (Stettler et al. 2009). This inconsistency may be explained by differences in growth stages and conditions between the 2 studies. Our study revealed that autophagy is not involved in leaf chlorosis in young *mex1* leaves but is strongly linked to the aggravation of leaf chlorosis in older *mex1* plants, suggesting that autophagy is not always active in *mex1* leaves. Perhaps autophagy is active at the growth stage when leaf chlorosis becomes exacerbated in *mex1* plants. The timing of this aggravation of chlorosis may largely depend on growth conditions. Therefore, changes in *ATG* transcript levels may closely reflect plant growth stages and conditions. Additionally, although we detected significant increases in the transcript levels of some *ATG* genes by RT-qPCR, no *ATG* genes were among the upregulated DEGs in *mex1* leaves as defined by RNA-seq analysis (Table S1). Therefore, the extent of changes in *ATG* transcript levels detected in large-scale transcriptome analyses such as microarrays and RNA-seq does not appear to be as drastic as those detected by RT-qPCR.

### Maltose hyperaccumulation might induce excessive chlorophagy

Autophagy is a vital process for removing unnecessary intracellular components that are damaged, aggregated, or present in excess. Therefore, the loss of core ATG proteins usually compromises proper cell functions, inhibiting plant growth and stress tolerance (Yagyu and Yoshimoto 2024). For instance, the leaves of *atg* mutants show accelerated cell death during senescence (Yoshimoto et al. 2009). Survivability under abiotic stress conditions such as heat or ultraviolet-B radiation is reduced in *atg* plants compared to WT plants (Izumi et al. 2017; Wan et al. 2023). However, the loss of ATG7 or ATG10 function partially rescued the leaf chlorosis and growth retardation caused by maltose hyperaccumulation (Fig. 7). These results indicate that maltose-excess stress within chloroplasts and the subsequent production of many swollen chloroplasts might cause the over-activation of autophagy including chlorophagy, accelerating chloroplast digestion and aggravating the leaf-chlorosis phenotype. Therefore, we suggest that key signals that induce chlorophagy are highly stimulated in the leaves of *mex1* plants as well as leaves damaged by HL. Common DEGs upregulated both by the mutation of *MEX1* and HL treatment potentially include the genes related to such signals (Fig. 8).

Of the WRKY and NAC transcription factors encoded by the commonly upregulated DEGs, WRKY46, WRKY51, WRKY60, WRKY62, and WRKY75 are thought to regulate SA biosynthesis and its downstream processes including autophagy (Zhang et al. 2017). Some transcription factors, including WRKY8, WRKY15, WRKY30, WRKY53, NAC016, and NAC042, were identified as candidates that directly bind to the promoter regions of *ATG8* genes (Wang et al. 2020). NAC046 and NAC087 redundantly regulate programmed cell death in root cap cells that are shed from the root tip (Huysmans et al. 2018). Autophagy is also required to facilitate this cell death in the root tip (Feng et al. 2022). Therefore, these NAC transcription factors might act as regulators of autophagy pathways related to the onset of programmed cell death. It is unclear if the WRKYs and NACs described in this study are involved in the activation of chlorophagy.

Maltose hyperaccumulates in the chloroplasts of *mex1* leaf mesophyll cells (Lu et al. 2006). Excess light energy from HL treatment causes ROS overproduction mainly within the chloroplast photosynthetic apparatus. Therefore, these 2 stress conditions likely occur inside the chloroplasts. Nevertheless, DEGs commonly upregulated by the mutation of *MEX1* and by HL treatment were enriched in key genes related to responses to external stimuli, such as bacterial infection and wounding (Fig. 8). A simple explanation for this phenomenon is that ROS accumulation is responsible for these transcriptional changes, since ROS themselves can trigger transcriptomic changes under various stress conditions, and ROS are well-known signals in plant defense to bacterial infection or local acclimation at the wounding site. For instance, the rapid production of ROS from oxygen (known as an oxidative burst) controls early responses to external stimuli (Mittler et al. 2022). ROS production due to strong light exposure induces the production of jasmonic acid and its precursors from phospholipids present in the chloroplast membrane (Kilic et al. 2025). Therefore, chloroplast-related ROS production due to the mutation of *MEX1* and HL treatment might cause changes in gene expression that partially overlap with responses to pathogen infection or wounding.

Another hypothesis is that damaged chloroplasts are recognized as components that must be eliminated in a manner similar to that used for foreign objects such as infecting organisms. Accordingly, responses to invasion by extracellular pathogens might be upregulated in response to chloroplast damage, thus inducing chlorophagy. In mammalian cells, bacteria invading host cells (eg, *Salmonella*) are engulfed by the endosomal membrane derived from the host cell membrane and are released into the cytoplasm when the surrounding membrane is ruptured (Vargas et al. 2023). The released bacteria are ubiquitinated and sequestered by autophagosomes via xenophagy (Perrin et al. 2004; Birmingham et al. 2006). The incorporation of polystyrene beads and subsequent rupture of the endosomal membrane stimulated the creation of autophagosome-like membranes around the beads, indicating that the rupture of the host cell membrane acts as a signal that induces xenophagy in mammalian cells (Fujita et al. 2013). Therefore, rupture or damage of the chloroplast envelope associated with the swelling phenotype might also serve as an activating signal for chlorophagy, and the signaling pathway might partially overlap pathways that function in plant responses to external stimuli. Further elucidation of the regulatory mechanisms underlying chlorophagy would provide deeper understanding of how changes in gene expression contribute to the induction of chlorophagy. Therefore, *mex1* plants represent powerful tools to help elucidate such a molecular mechanism.

## Materials and methods

### Plant materials and growth conditions

The Arabidopsis (*A. thaliana*) accession Columbia (Col-0) was used as the WT for all experiments. The Arabidopsis T-DNA insertion lines *mex1-3* (SAIL_574_D11), *atg5-1* (SAIL_129_B07), *atg7-2* (GABI_655B06), and *atg10-1* (SALK_084434) were described previously (Thompson et al. 2005; Phillips et al. 2008; Hofius et al. 2009; Izumi et al. 2010). The EMS-generated point mutants of *PGM* (*pgm-1*) and *SEX1* (*sex1-1*) were described previously (Caspar et al. 1985, 1991). Seeds of the mutant alleles *mex1-1* (CS2105661; Niittylä et al. 2004) and *mex1-4* (SALK_201638) were obtained from the Arabidopsis Biological Resource Center. Double mutant lines were generated by genetic crossing. Transgenic Arabidopsis lines carrying a transgene encoding a chloroplast stroma–targeted GFP driven by the cauliflower mosaic virus (CaMV) *35S* promoter (*35S:CT-GFP*), RBCS2B-mRFP driven by the *RBCS2B* promoter (*ProRBCS:RBCS-mRFP*), TOC64-mRFP driven by the *TOC64* promoter (*ProTOC64:TOC64-mRFP*), and GFP-ATG8a driven by the *UBQ10* promoter (*ProUBQ:GFP-ATG8a*) were previously described (Kohler et al. 1997; Nakamura et al. 2021b; Izumi et al. 2024).

To generate transgenic plants accumulating a fluorescently labeled vacuolar membrane marker, the full-length coding sequence of *δTIP* (AT3G16240), which was previously cloned into the pENTR/D-TOPO entry vector (Kusano et al. 2022), was recombined into the pUBC-YFP-Dest vector (Grefen et al. 2010) via an LR Clonase II reaction (Invitrogen). To generate transgenic plants accumulating a fluorescently labeled chloroplast outer envelope marker, the genomic fragment of *TOC64-III* (AT3G17970), which was previously cloned into the pDONR221 entry vector (Kusano et al. 2023), was recombined into the pGWB504 vector (Nakagawa et al. 2007). To generate transgenic plants producing NBR1-GFP, the full-length coding sequence of *NBR1* (AT4G24690) was amplified from Arabidopsis Col-0 cDNA using the primers described in Table S7, cloned into the pENTR1A entry vector (Invitrogen) by a NEBuilder HiFi DNA Assembly Master Mix reaction (NEB), and transferred into the pUBC-GFP-Dest vector (Grefen et al. 2010) via an LR Clonase II reaction. All vectors were introduced into Agrobacterium (*Agrobacterium tumefaciens*) strain GV3101 before being introduced into Arabidopsis plants by the floral dip method (Clough and Bent 1998).

Plants were grown in soil in chambers (LH-411PFDT-SP; Nippon Medical & Chemical Instruments) set to 23 °C under a 14-h light/10-h dark photoperiod with LEDs (120 to 140 μmol m^−2^ s^−1^). The second rosette leaves of 14-d-old seedlings and the third rosette leaves of 21-d-old and 28-d-old plants were used to measure the maximum quantum yield of PSII (*F*_v_/*F*_m_) and for confocal microscopy.

### Measurement of the quantum yield of PSII

The maximum quantum yield of PSII (*F*_v_/*F*_m_) was calculated using a pulse-modulated fluorometer (FluorCam FC 800; Photon Systems Instruments) at room temperature. Before measuring fluorescence emission, the plants were incubated in the dark for 30 min. The initial (minimum; *F*_0_) and maximum PSII fluorescence (*F*_m_) in the dark-adapted state were measured with a measuring light and a saturating pulse, from which *F*_v_/*F*_m_ was calculated, with *F*_v_ = *F*_m_ − *F*_0_.

### Confocal laser scanning microscopy and image analysis

Confocal laser scanning microscopy (CLSM) was performed with an inverted Nikon C2 system equipped with a CFI Apochromat LWD λS 40×C WI (numerical aperture, 1.15). Emissions were collected between 500 and 550 nm (bandpass filter RPB500–550; Omega optical) for GFP and between 660 and 720 nm for chlorophyll fluorescence following excitation with a 489.6-nm diode laser. Emission was collected from 580 to 630 nm (bandpass filter RPB580–630; Omega optical) for RFP after excitation with a 559.8-nm diode laser and from 660 to 720 nm for chlorophyll fluorescence following excitation with a 636.5-nm diode laser.

To quantitatively evaluate the frequency of chlorophagy, 3 different regions (each 273.1 µm × 273.1 µm × 15 μm) in a leaf (the second rosette leaf from a 14-d-old seedling, or the third rosette leaf from a 21-d-old or 28-d-old plant) were monitored by adjusting the focus to calculate the mean proportion of cells with vacuole-enclosed chloroplasts. This observation was performed in 4 independent plants. For the colocalization assay between stromal GFP and chlorophyll fluorescence (Figs. 3f and 5b), *z*-stack images of 2 different regions (each 102.4 µm × 102.4 µm × 10 µm) in a leaf were examined by CLSM. The colocalization ratio between the GFP signal and the chlorophyll signal was obtained using Imaris software (Oxford Instruments), and the mean value was calculated. This observation was performed in 4 independent plants. To quantitatively evaluate mRFP accumulation in the vacuole (Figures S4 and S7), the RFP fluorescence intensities in 8 or 10 different vacuoles were measured in a leaf, and the mean intensity was calculated for each of nine or six individual plants, respectively, as indicated in the figure legends. To count the accumulated RCBs (Figure S9), the third rosette leaves of 21-d-old plants were excised, infiltrated with 10 mM MES-NaOH, pH 5.5, containing 0.5 μM concanamycin A (sc-202111; Santa Cruz), and incubated for 1 d in the dark at 23 °C. The number of accumulated RCBs in 4 different areas (each 317.44 µm × 317.44 µm) was counted in a leaf, and the mean value was calculated. This observation was performed in 4 independent plants.

GFP-ATG8a signals (Fig. 6), NBR1-GFP signals (Figure S11), and the materials shown in Figures S1, S2, S10, and S16 were observed using a Zeiss LSM900 system equipped with a C-Apochromat 40x/1.20 W Korr lens. Emissions were collected between 410 and 546 nm for GFP and between 656 and 700 nm for chlorophyll fluorescence following excitation with a 488-nm diode laser, or between 550 and 640 nm for RFP following excitation with a 561-nm laser. To quantify chloroplasts associated with flattened GFP-ATG8a signals or chloroplasts accumulating NBR1-GFP-labeled puncta, 5 different regions (each 226.21 µm × 226.21 µm × 20 µm) in a leaf were monitored to calculate the sum of each type of chloroplast. This observation was performed in 4 individual plants. The number of extended globular structures of chloroplasts was calculated as the mean in a given region (each 159.73 µm × 159.73 µm × 20 µm) from observations of 4 regions. These observations were performed in 4 individual plants.

### Electron microscopy observations

The third rosette leaves of 21-d-old plants were cut into small pieces (∼2 to 3 mm wide) and fixed overnight at 4 °C in fixation solution containing 2% (v/v) glutaraldehyde and 4% (w/v) formaldehyde in 50 mM sodium cacodylate buffer, pH 7.4. Following fixation, the samples were washed with the same buffer and post-fixed with 1% (w/v) osmium tetroxide in 50 mM cacodylate buffer for 3 h at room temperature. The samples were dehydrated through a graded methanol series (25%, 50%, 75%, 90%, 100%, all v/v), infiltrated with resin using increasing concentrations of Epon812 resin (T024; TAAB) mixed with propylene oxide (the ratios of propylene oxide to Epon812; 3:1, 1:1, 1:3, all v/v), and embedded in 100% Epon812. Polymerized resin blocks were sectioned using an ultramicrotome (Leica Microsystems EM UC7) equipped with a diamond knife (Histo or Ultra, Diatome). Ultrathin sections (100 nm thick) were mounted onto glass slides. Sections were stained sequentially with 0.4% (w/v) uranyl acetate for 12 min and then lead stain solution (18-0875; Sigma-Aldrich) for 3 min. Prior to electron microscopy, the sections were coated with osmium using an osmium coater (HPC-1SW; Vacuum Device) for 3 s. Imaging was performed using a field emission scanning electron microscope (FE-SEM, Hitachi High-Tech SU8220) equipped with a yttrium aluminum garnet backscattered electron detector (YAG-BSE) or a Hitachi High-Tech SU8600 equipped with an out column crystal type BSE detector (OCD-BSE), operated at 5 kV.

### Immunoblotting

For the cleavage assay of RBCS-mRFP, leaves were homogenized in HEPES-NaOH (pH 7.5) containing 16 mM DTT, 10% (v/v) glycerol, and protease inhibitor cocktail (04080-11; Nacalai). The mixture was centrifuged at 20,630 *g* for 10 min at 4℃. The protein concentrations in the supernatants (soluble protein fraction) were quantified using a 660-nm Protein Assay Reagent (Pierce). An aliquot of the soluble protein fraction was mixed with an equal volume of SDS sample buffer consisting of 200 mM Tris-HCl (pH 8.5), 2% (w/v) SDS, 0.1 M DTT, and 20% (v/v) glycerol and incubated for 5 min at 95℃. Equal amounts of protein were separated by SDS-PAGE using TGX FastCast acrylamide gels (Bio-Rad) before being transferred to nitrocellulose membranes (Trans-blot turbo transfer pack; Bio-Rad). Immunoblotting was performed with an anti-RFP 1G9 clone antibody (1:2,000, M204-3; MBL) and an anti-cFBPase antibody (1:5,000, AS04043; Agrisera). Goat anti-mouse IgG (H + L) secondary antibody DyLight800 4 × PEG (1:10,000, SA5-35521; Invitrogen) and goat anti-rabbit HRP secondary antibody (1:10,000, NA934; Cytiva) were used as the secondary antibodies. The chemiluminescence signals developed with SuperSignal West Dura Extended Duration Substrate (Pierce) and DyLight 800 fluorescent signals were detected using a ChemiDoc MP system (Bio-Rad). Image processing and the quantification of band intensity were performed using Image Lab Software (Bio-Rad).

### Quantification of carbohydrate content

Sugar and starch fractions were extracted from the samples as previously described (Izumi et al. 2010), with slight modifications. The third and fourth rosette leaves from 21-d-old plants were harvested at the end of the night to quantify maltose content or in the middle of the light period (7 h after dawn) to quantify starch content. The leaves from 2 plants were pooled as one sample. The leaves were rapidly frozen in liquid N_2_ and homogenized in a liquid N_2_-chilled tube with a TissueLyser (QIAGEN) and a zirconium bead. After adding 25 nmol D-Glucose ^13^C_6_ (Santa Cruz) to each sample as a normalization standard, the sample was incubated in 0.8 mL of 80% (v/v) ethanol at 80 °C for 12 min. The mixture was centrifuged at 20,630 *g* for 5 min at room temperature and the supernatants collected. This treatment was repeated 3 more times on the pellet residue. The individual supernatants were combined and concentrated with a centrifugal concentrator (VC-96R; Taitec) equipped with a freeze trap (VA-500R; Taitec). The dried residue was dissolved in 50% (v/v) acetonitrile, and the resuspended extracts were filtered through a C-18 Empore disc (CDS Analytical). The concentrations of maltose, glucose, and sucrose in the filtrates were measured using a Vanquish UHPLC and TSQ Altis system (Thermo Fisher Scientific) with an electrospray ionization ion source in negative mode at the Support Unit for Bio-Material Analysis of the RIKEN Center for Brain Science, Research Resources Division. The compounds were separated in a BEH-amide column (2.1 × 50 mm, 1.7 µm, Waters) at 40 °C using isocratic elution with 13% (v/v) solvent A (10% [v/v] acetonitrile and 0.1% [v/v] NH_4_OH) and 87% (v/v) solvent B (90% [v/v] acetonitrile and 0.1% [v/v] NH_4_OH) for 7.5 min at a flow rate of 0.17 mL/min. Target compounds were detected in multiple-reaction monitoring (MRM) mode with the following transitions: glucose (*m/z* 89 to 179), sucrose (*m/z* 89 to 341), maltose (*m/z* 161 to 341). TraceFinder software (Thermo Fisher Scientific) was used for quantification.

The ethanol-insoluble fraction was dried overnight at room temperature. Starch in this fraction was extracted with 0.6 mL of dimethyl sulfoxide (DMSO) and 0.15 mL of 8 M HCl at 60 °C for 30 min with gentle shaking (600 rpm in ThermoMixer C; Eppendorf). The pH was then neutralized to between 4.0 and 5.0 with 0.1 mL of 2 M sodium acetate buffer (pH 5.5) and 8 M NaOH. Each solution was adjusted to a final volume of 1.0 mL with ultrapure water. The starch concentration was determined using a Starch Assay Kit (SA20, Sigma-Aldrich) and an Infinite 200 PRO plate reader (Tecan). Glucose (Sigma-Aldrich) was used as the standard, and the starch contents equivalent to glucose are shown.

### RT-qPCR

Total RNA was isolated from rosette leaves using a Maxwell RSC Plant RNA Kit (Promega) and a Maxwell RSC instrument (Promega). The third rosette leaves from 2 plants were pooled as a sample. Equal amounts of total RNA were subjected to reverse transcription with PrimeScript RT Master Mix (Takara). An aliquot of the first-strand cDNA was subjected to qPCR analysis using standard curves with SsoAdvanced Universal SYBR Green Supermix (Bio-Rad) on a real-time PCR detection system (CFX96, Bio-Rad). The level of *18S* rRNA was measured as an internal control. The primer sequences for RT-qPCR are listed in Table S7.

### Measurement of chloroplast number

The number of chloroplasts in mesophyll cells was counted as previously described (Pyke and Leech 1991), with slight modifications. Fragments of the third rosette leaves from 21-d-old plants were excised and infiltrated with 3.5% glutaraldehyde in a 10-mL syringe by negative pressure caused by pulling the plunger, followed by 5 min of incubation in the dark. The infiltrated leaf fragments were washed twice with 0.1 M EGTA, pH 8.0, and incubated in the same buffer for 2.5 h at 60 °C with gentle shaking. The fixed leaf fragments were pressed with a cover glass on a glass slide to disperse individual cells and observed through an inverted-type Nikon ECLIPSE Ti2 microscope in a Nikon C2 system. Chloroplast number was counted in 10 cells of typical size in each genotype in 4 independent plants. Bright-field images and chlorophyll fluorescence of the fixed cells were obtained using the Nikon C2 system.

### Transcriptome deep sequencing (RNA-seq)

Two experimental settings were prepared for RNA-seq. The third and fourth rosette leaves from 21-d-old WT, *mex1-3*, and *sex1-1* plants were used for one set, while the other set consisted of second rosette leaves or leaves 1 d after HL exposure from 15-d-old WT plants. The leaves from two 21-d-old plants or four to five 15-d-old plants were pooled as one sample, respectively. For HL treatment, soil-grown plants at 14 d after sowing were exposed to intense visible light (2,000 µmol m^−2^ s^−1^) emitted from a Xenon light source (MAX-303; Asahi Spectra) equipped with a mirror module (MAX-VIS; Asahi Spectra) and a rod lens (RLQL80-1; Asahi Spectra) for 2 h in an incubator, followed by 1 d of incubation under normal growth conditions because vacuole-enclosed chloroplasts transported by chlorophagy start to appear from 1 d after HL treatment (Nakamura et al. 2018). Total RNA was isolated from the samples as described above. Total RNA concentrations were measured using a QuantiFluor RNA System (Promega). Equal amounts of total RNA (400 ng) were used as input for library preparation using the Lasy-Seq method (Kamitani et al. 2019) version 1.1 (https://sites.google.com/view/lasy-seq/). The libraries were sequenced using a HiSeq X instrument (Illumina) as 150-bp paired-end reads. The forward reads were processed using the quality trimming software fastp (Chen et al. 2018) to 126-bp reads and mapped to the Arabidopsis reference sequence (Araport11) using STAR (Dobin et al. 2013). The mapped reads were counted with featureCounts (Liao et al. 2014). Genes that mapped to the genomes of organelles and those with low counts (with a mean count among all samples <1) were excluded from the count dataset prior to subsequent analysis.

The count dataset was normalized with the R package DESeq2 (Love et al. 2014), and DEGs were identified from comparisons between the leaves of 21-d-old *mex1* and WT plants and between leaves 1 d after high-light treatment and untreated control leaves from 15-d-old seedlings. Genes with an absolute log_2_(fold change) ≥ 1 and adjusted *P* < 0.01 from the DESeq2 analysis were defined as differentially expressed. Hierarchical clustering and heatmaps of DEGs were produced by the R packages ggdendro and ggplot2 (Wickham 2016). GO enrichment analysis was performed with the R packages clusterProfiler (Xu et al. 2024) and org.At.tair.db.

### Statistical analysis

Statistical analysis was performed with JMP software (SAS Institute). Student's *t*-test was used to compare paired samples, while one-way ANOVA and Tukey's test were used to compare multiple samples, as indicated in the figure legends.

### Accession numbers

The RNA-seq data from this article can be found in the DDBJ database under BioProject accession number PRJDB37410. Sequence data from this article can be found in the Arabidopsis Information Resource database under the following accession numbers: *ADG1*, AT5G48300; *ATG5*, AT5G17290; *ATG7*, AT5G45900; *ATG8a*, AT4G21980; *ATG8b*, AT4G04620; *ATG8c*, AT1G62040; *ATG8d*, AT2G05630; *ATG8e*, AT2G45170; *ATG8f*, AT4G16520; *ATG8g*, AT3G60640; *ATG8h*, AT3G06420; *ATG8i*, AT3G15580; *ATG9*, AT2G31260; *ATG10*, AT3G07525; *BAM1*, AT3G23920; *BAM3*, AT4G17090; *BE2*, AT1G03310; *BE3*, AT2G36390; *GBSS1*, AT1G32900; *MEX1*, AT5G17520; *NAC016*, AT1G34180; *NAC042*, AT2G43000; *NBR1*, AT4G24690; *PGM*, AT2G17280; *RBCS2B*, AT5G38420; *SEX1*, AT1G10760; *SEX4*, AT3G52180; *SS1*, AT5G24300; *SS2*, AT3G01180; *SS3*, AT1G11720; *SS4*, AT4G18240; *TOC64-III*, AT3G17970; *WRKY16*, AT5G45050; *WRKY62*, AT5G01900; and *δTIP*, AT3G16240.

## Supplementary Material

kiag271_Supplementary_Data

## Data Availability

The RNA-seq data underlying this article have been deposited in the DDBJ database (https://www.ddbj.nig.ac.jp/index-e.html) under accession number PRJDB37410. The raw data underlying the figures are available from the corresponding author upon reasonable request.
