# Supplementary material for: Chloroplast stress caused by maltose hyperaccumulation activates chlorophagy via the core autophagy machinery
Source: Plant Physiol. 2026 May 8;201(1):kiag271. doi: 10.1093/plphys/kiag271 (PMC13222026; doi:10.1093/plphys/kiag271)
Supplement: kiag271_Supplementary_Data [file kiag271_supplementary_data.zip › SupplementaryFigures_updated_2.pdf]

Supplementary Figure S1

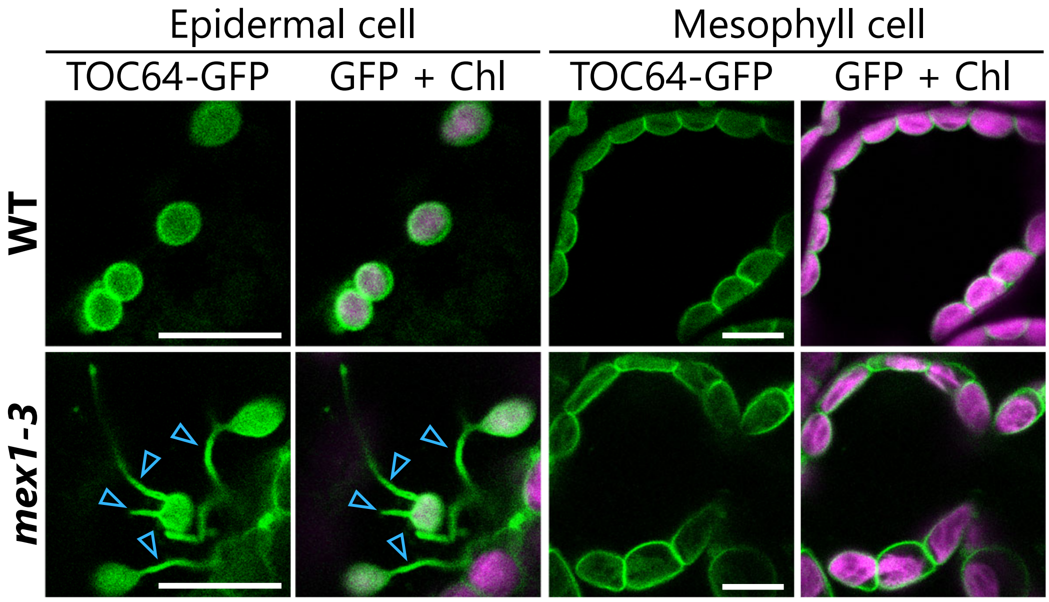

Supplementary Figure S1. Observation of stromules in epidermal cells in *mex1* leaves.
Representative confocal microscopy images of chloroplasts in epidermal cells or mesophyll cells from the leaves of 21-d-old WT and *mex1-3* Arabidopsis plants producing the chloroplast outer envelope membrane protein TOC64-GFP. Green, TOC64-GFP; magenta, chlorophyll fluorescence (Chl). Arrowheads, stromules. Scale bars, 10 μm.

Supplementary Figure S2

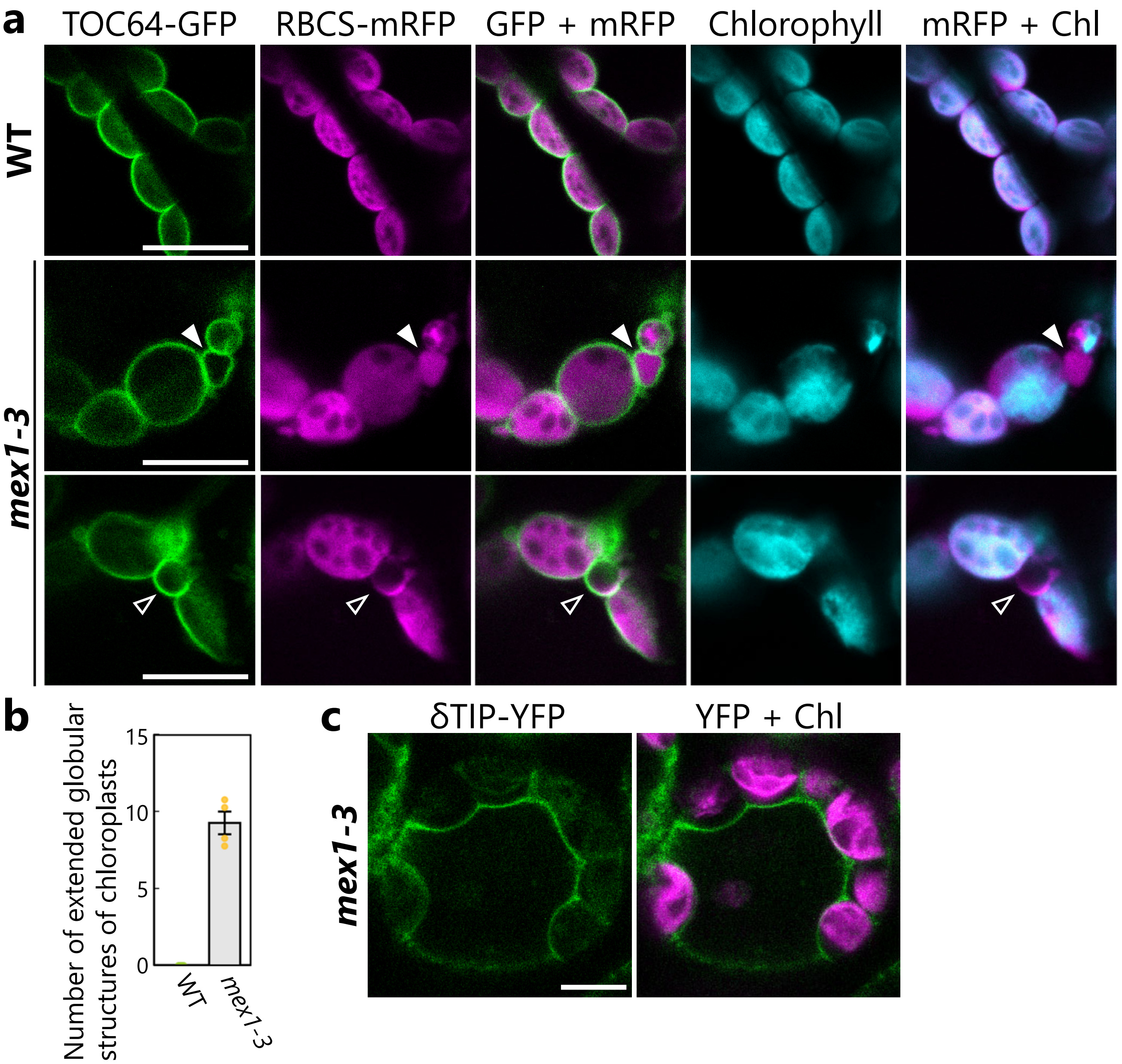

Supplementary Figure S2. Additional observations of the extended globular structures of chloroplasts in *mex1-3* leaves.

a) Confocal microscopy images of chloroplasts from leaf mesophyll cells of 21-d-old WT and *mex1-3* Arabidopsis plants producing TOC64-GFP and the chloroplast stroma marker RBCS-mRFP. Representative images of the extended globular structures of chloroplasts are shown in the images from *mex1-3* leaves. Green, TOC64-GFP; magenta, RBCS-mRFP; cyan, chlorophyll fluorescence (Chl). Filled arrowheads, globular structures containing RBCS-mRFP; open arrowheads, globular structures without RBCS-mRFP. Scale bars, 10 µm.

b) Number of extended globular structures of chloroplasts in a given region based on the observations described in (**a**). The bars indicate means ± SE from four individual plants (*n* = 4).

c) Representative confocal microscopy images of leaf mesophyll cells from 21-d-old *mex1-3* plants accumulating the tonoplast marker δTIP-YFP. Green, δTIP-YFP; magenta, chlorophyll fluorescence (Chl). Scale bars. 10 µm.

Supplementary Figure S3

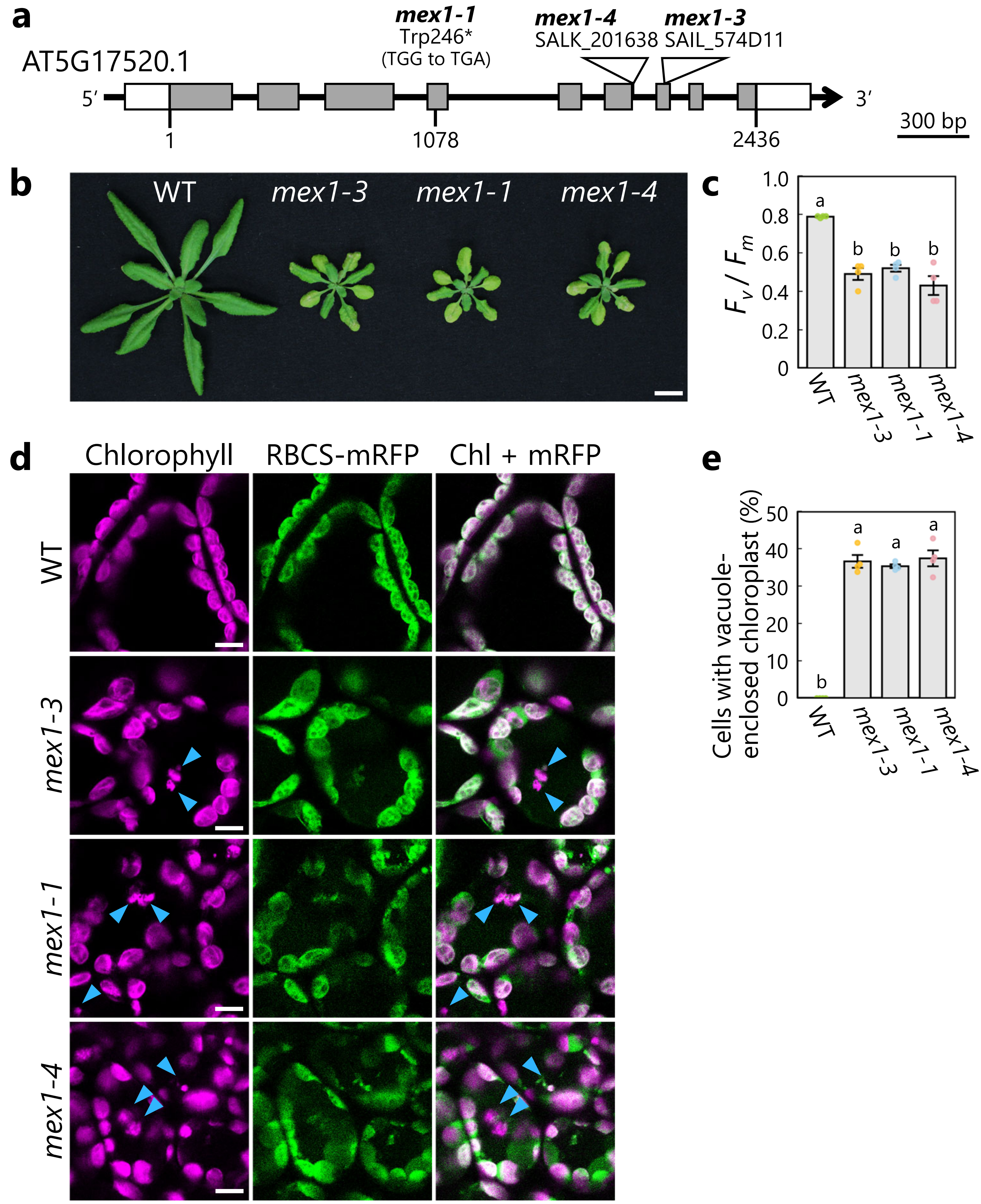

Supplementary Figure S3. Multiple *mex1* mutant alleles undergo chloroplast degradation in the vacuole.

a) Diagram of the *MEX1* (AT5G17520.1) locus. The positions of the T-DNA insertions in *mex1-3* (SALK_574_D11) and *mex1-4* (SALK_201638) and the point mutation (TGG to TGA, introducing a stop codon in place of Trp-246) in *mex1-1* are shown. White boxes, gray boxes, and black lines between gray boxes, represent untranslated regions, exons, and introns, respectively.

b) Representative photographs of wild type Col-0 (WT), *mex1-3*, *mex1-1*, and *mex1-4* plants grown in soil for 21 d. Scale bar, 10 mm.

c) F_v/F_m values measured from the third rosette leaves of the genotypes described in (**b**). Values are means ± SE from four independent plants (n = 4).

d) Representative confocal images of leaf mesophyll cells from 21-d-old WT, *mex1-3*, *mex1-1*, and *mex1-4* plants harboring the stromal marker transgene *RBCS-mRFP*. Magenta, chlorophyll fluorescence (Chl); green, RBCS-mRFP. Arrowheads, vacuole-enclosed chloroplasts. Scale bars, 10 µm.

e) Proportion of cells with vacuole-enclosed chloroplasts based on the confocal observations described in (**d**). The bars indicate means ± SE from four individual plants (n = 4).

In (**c**) and (**e**), dots represent data points from individual plants; different letters denote significant differences based on Tukey's test (P < 0.05).

Supplementary Figure S4

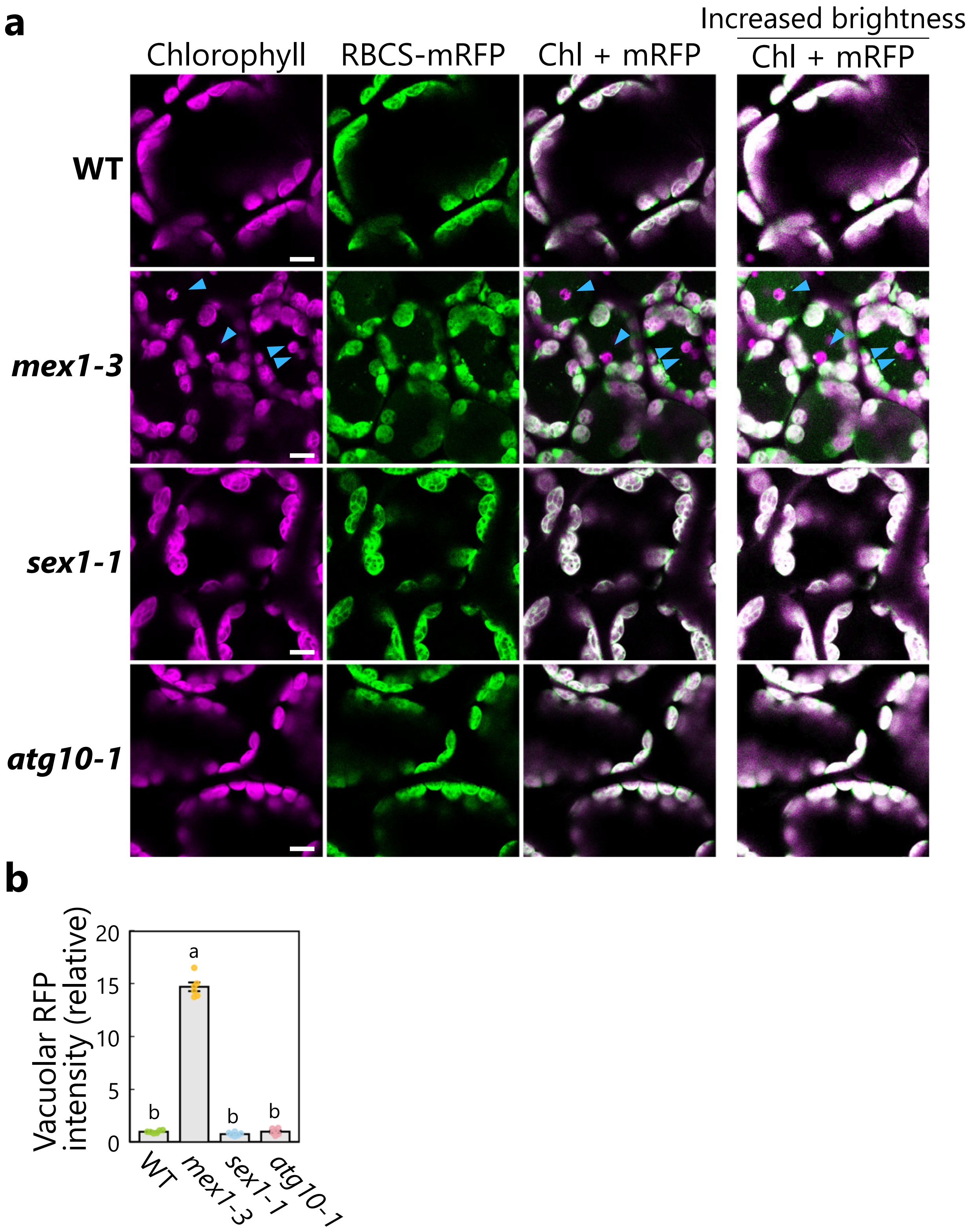

Supplementary Figure S4. The leaves of *mex1* plants accumulate a chloroplast stroma–targeted marker in the vacuole.

a) Representative confocal images of leaf mesophyll cells from 21-d-old wild-type Col-0 (WT), *mex1-3*, *sex1-1*, and *atg10-1* plants harboring the stromal marker transgene *RBCS-mRFP*. Magenta, chlorophyll fluorescence (Chl); green, RBCS-mRFP. Arrowheads, vacuole-enclosed chloroplasts. Scale bars, 10 μm.

b) RFP fluorescence intensity values from the vacuoles of leaf mesophyll cells observed in (**a**) relative to that in WT leaves, which was set to 1. The bars indicate means ± SE from six individual plants (n = 6). Dots represent data points from individual plants; different letters denote significant differences based on Tukey's test ($P < 0.05$).

Supplementary Figure S5

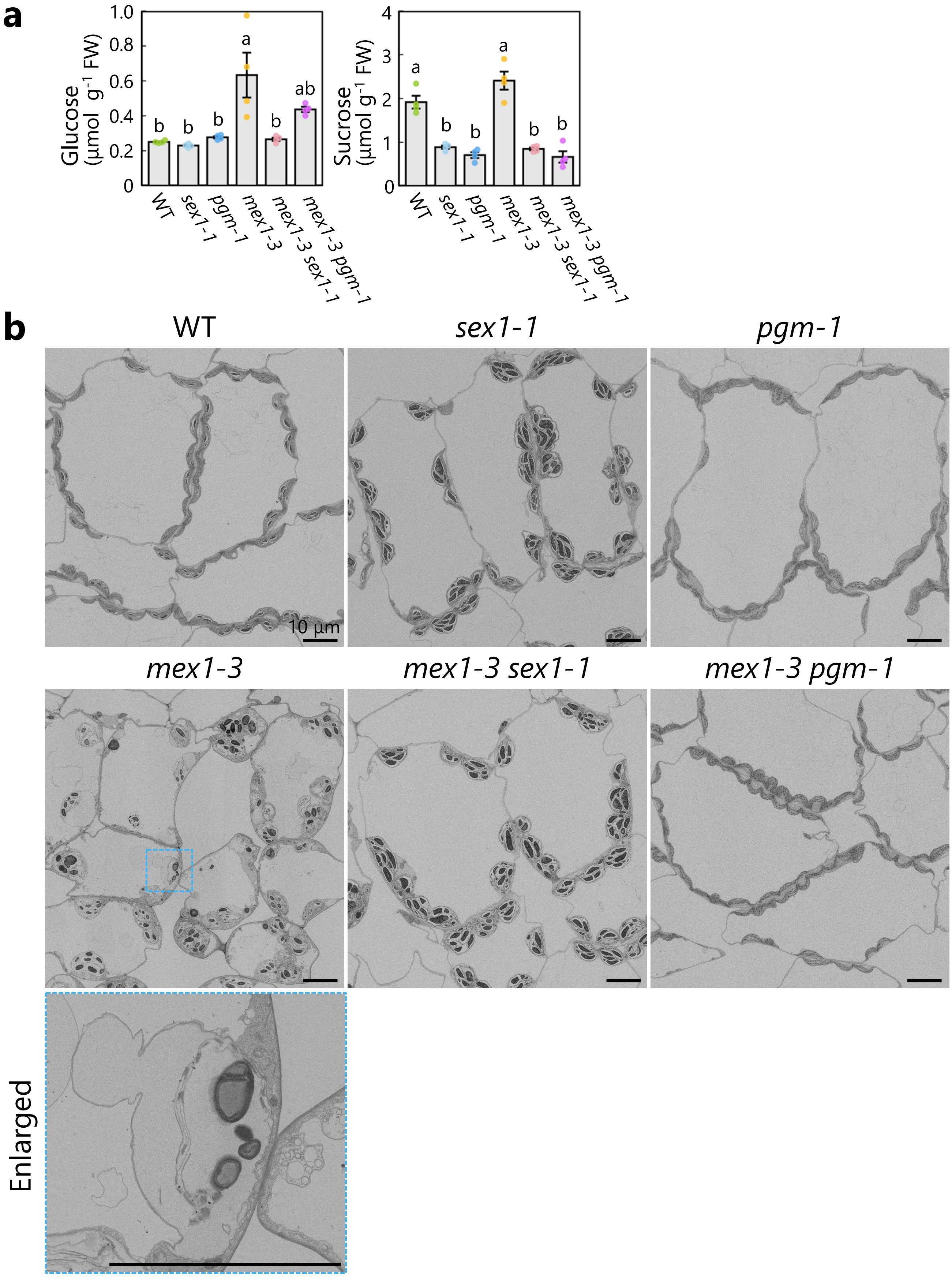

Supplementary Figure S5. Glucose and sucrose contents, and additional electron micrographs of the samples described in Figure 3.

a) Glucose and sucrose contents in leaf extracts described in Figure 3a. Values are means ± SE from four individual samples (n = 4). Dots represent data points from individual samples; different letters denote significant differences based on Tukey's test ($P < 0.05$).

b) Additional electron micrographs showing wide fields of leaf samples described in Figure 3e. The *sex1-1* image contains the region that appear as the enlarged image in Figure 3e. The area indicated by a dashed blue box in *mex1-3* image shows the chloroplast forming an extended globular structure, which is expanded as an enlarged image. Scale bars, 10 µm.

Supplementary Figure S6

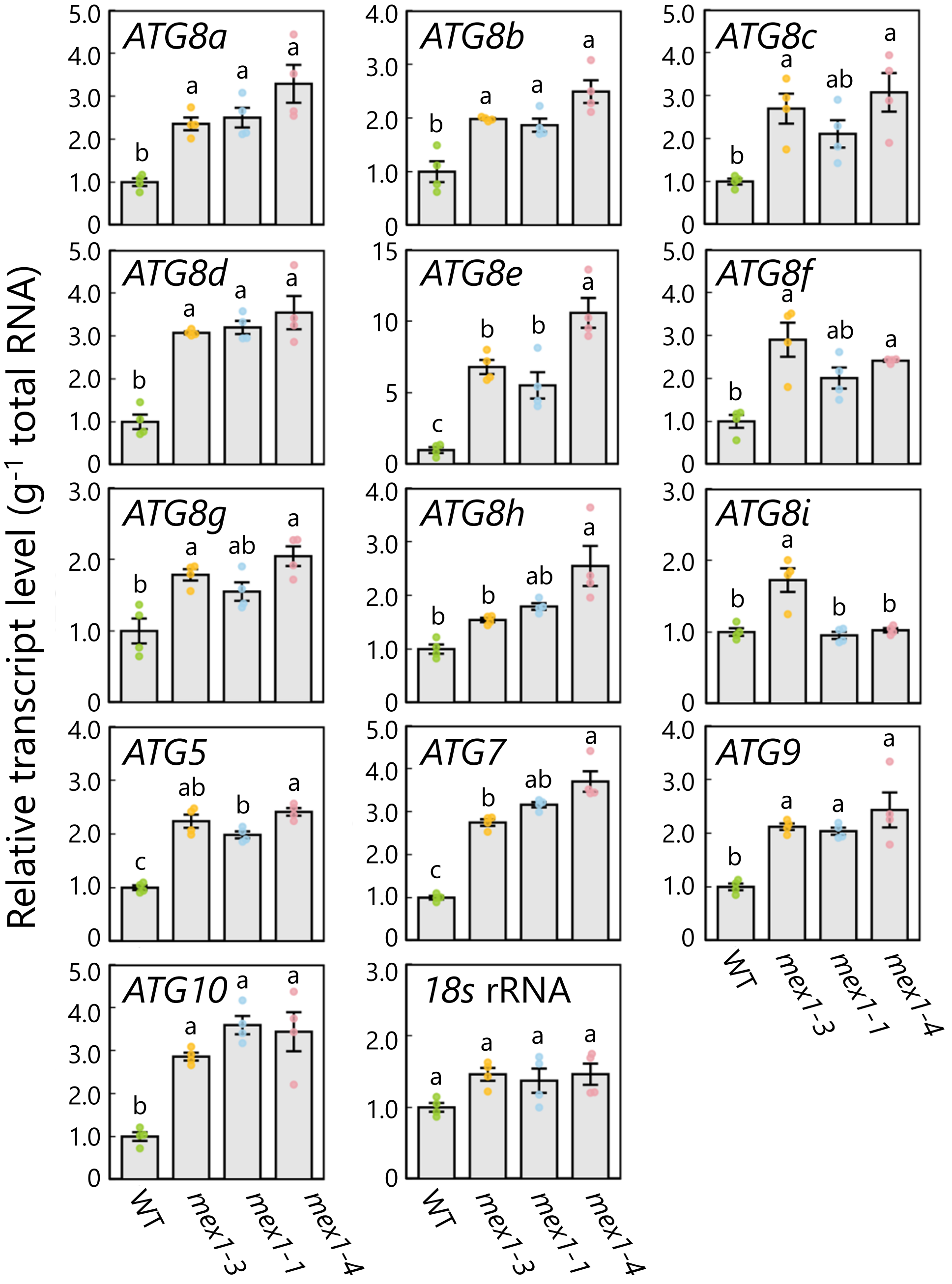

Supplementary Figure S6. ***ATG*** **genes are upregulated in multiple** ***mex1*** **alleles.**
Relative transcript levels of the autophagy-related genes *ATG8a–i*, *ATG5*, *ATG7*, *ATG9* and *ATG10* in the leaves of 21-d-old wild-type Col-0 (WT), *mex1-3*, *mex1-1*, and *mex1-4* plants relative to the values in WT leaves, which were set to 1. The level of *18S* rRNA was measured as an internal control. The bars indicate mean transcript levels ± SE from four individual samples (n = 4). In each plot, dots represent data points from individual samples; different letters denote significant differences based on Tukey's test (P < 0.05).

Supplementary Figure S7

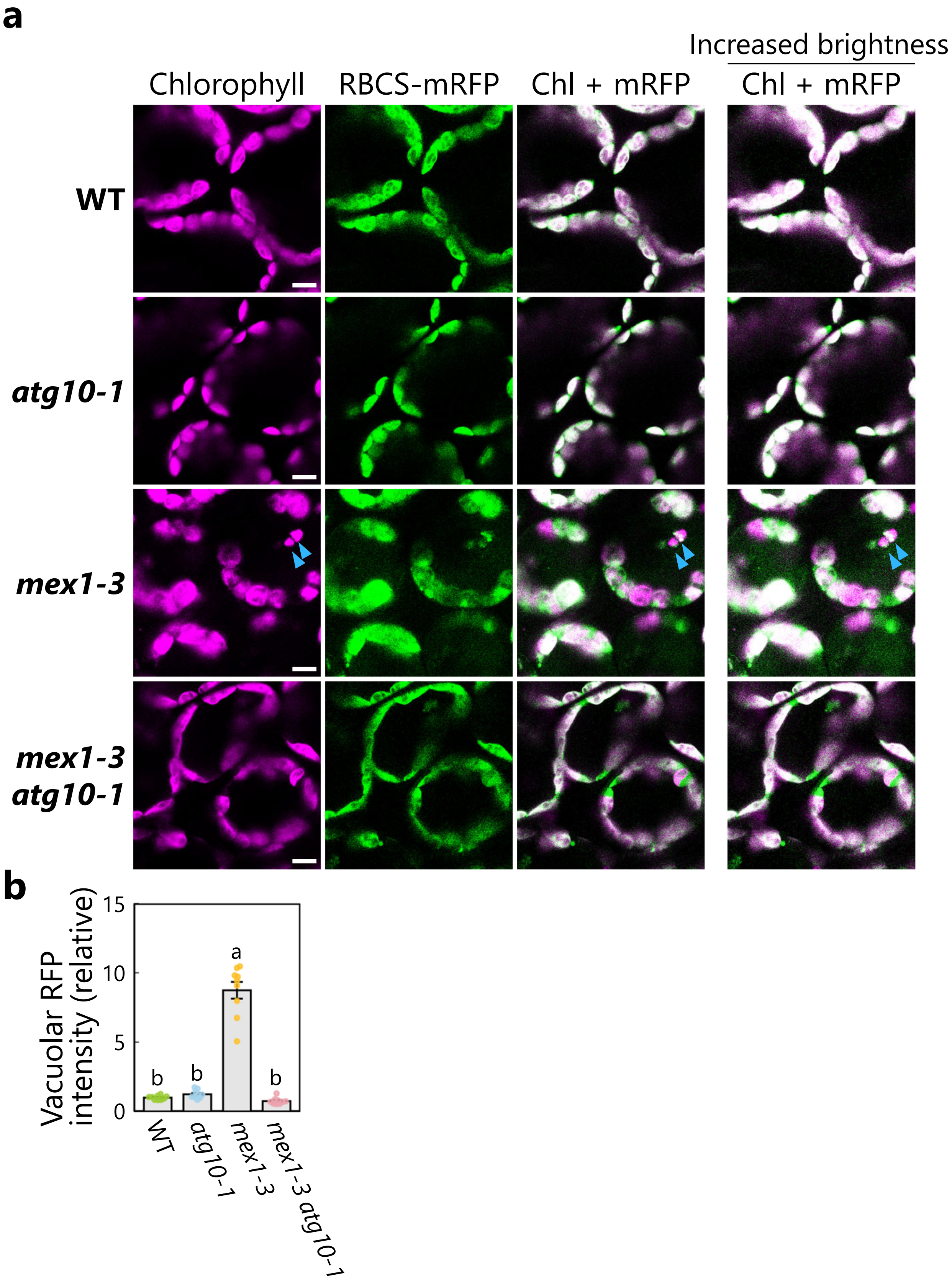

Supplementary Figure S7. The vacuolar accumulation of the chloroplast stroma–targeted fluorescent marker protein RBCS-mRFP in *mex1* leaves is suppressed by the loss of ATG10.

a) Representative confocal images of leaf mesophyll cells from 21-d-old wild-type Col-0 (WT), *atg10-1*, *mex1-3*, and *mex1-3 atg10-1* plants harboring the *RBCS-mRFP* transgene. Green, RBCS-mRFP; magenta, chlorophyll fluorescence (Chl). Arrowheads, vacuole-enclosed chloroplasts. Scale bars, 10 µm.

b) RFP fluorescence intensity in the vacuoles observed in (**a**) relative to that in WT leaves, which was set to 1. The bars indicate means ± SE from nine individual plants (n = 9). Dots represent data points from individual plants; different letters denote significant differences based on Tukey's test (P < 0.05).

Supplementary Figure S8

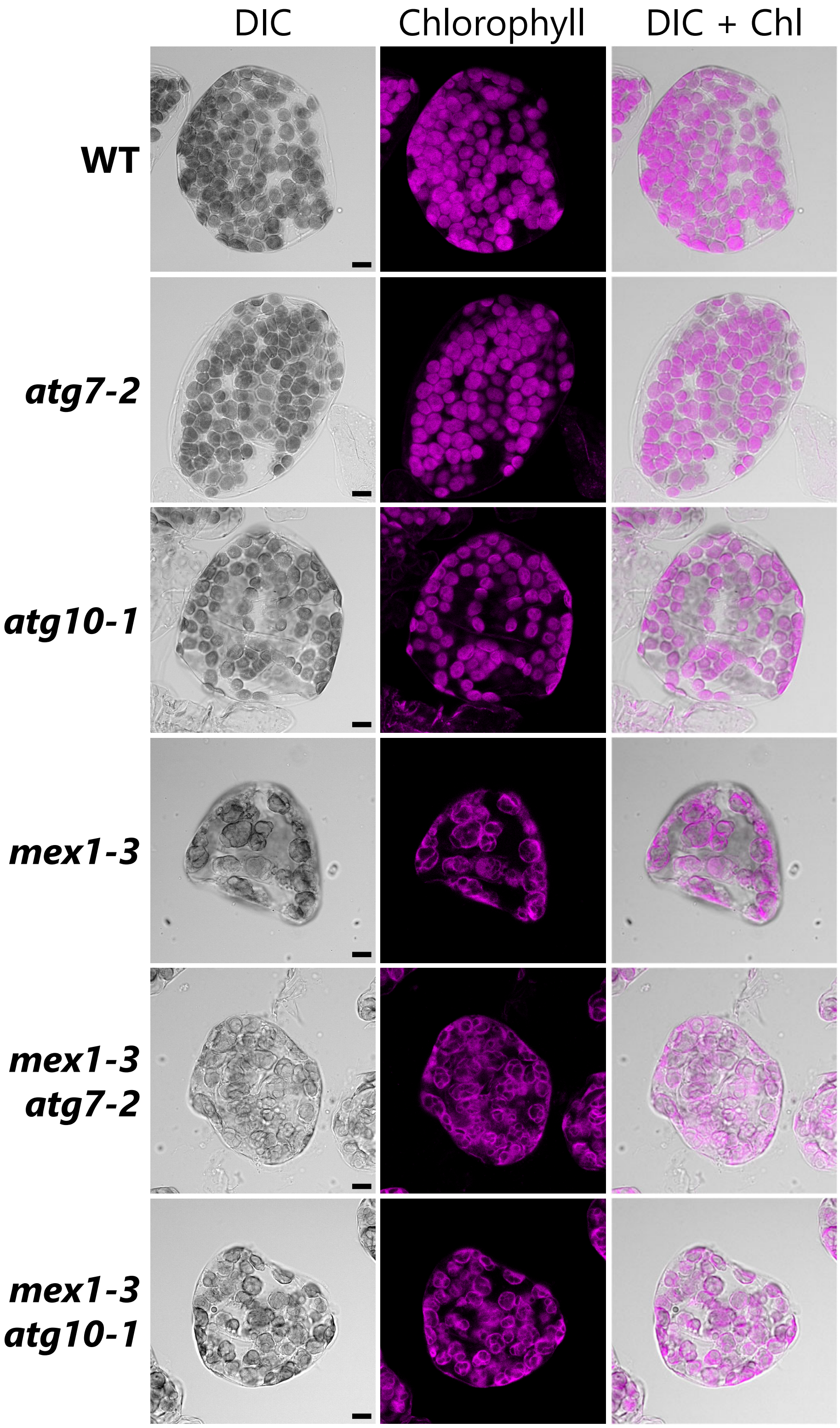

Supplementary Figure S8. Images of chemically fixed mesophyll cells used to measure chloroplast number shown in Figure 5F.
Representative confocal images of chemically fixed mesophyll cells from the leaves described in Figure 5f. DIC indicates differential interference contrast images. Magenta, chlorophyll fluorescence (Chl). Scale bars, 10 μm.

Supplementary Figure S9

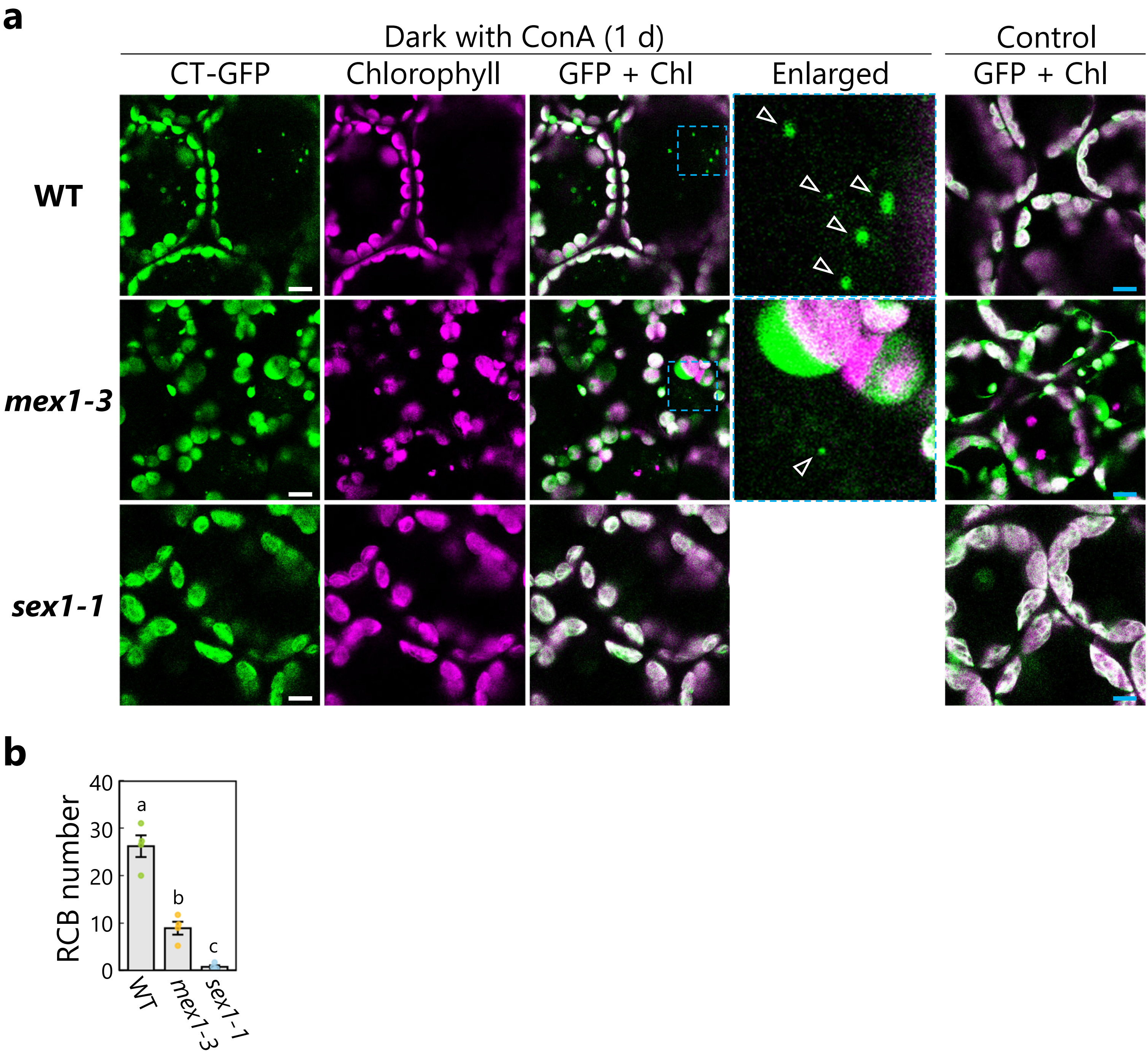

Supplementary Figure S9. Macroautophagy of chloroplast stromal components via Rubisco-containing bodies is not activated in the leaves of *mex1* plants.

a) Representative confocal images of mesophyll cells from leaves of plants harboring the *CT-GFP* transgene following incubation with 0.5 µM concanamycin A (ConA) for 1 d in the dark. Third rosette leaves from 21-d-old wild-type Col-0 (WT), *mex1-3*, and *sex1-1* plants were used. Untreated leaves were observed as control. Green, CT-GFP; magenta, chlorophyll fluorescence (Chl). The area indicated by a dashed blue box is expanded to the right of each panel as enlarged images, and the small GFP fluorescent dots indicated by arrowheads represent Rubisco-containing bodies (RCBs) in the vacuole. Scale bars, 10 µm.

b) Number of RCBs present in the vacuoles of the indicated genotypes based on the observations described in (**a**). The bars indicate means ± SE from four individual plants (n = 4). Dots represent data points from individual plants; different letters denote significant differences based on Tukey's test ($P < 0.05$).

Supplementary Figure S10

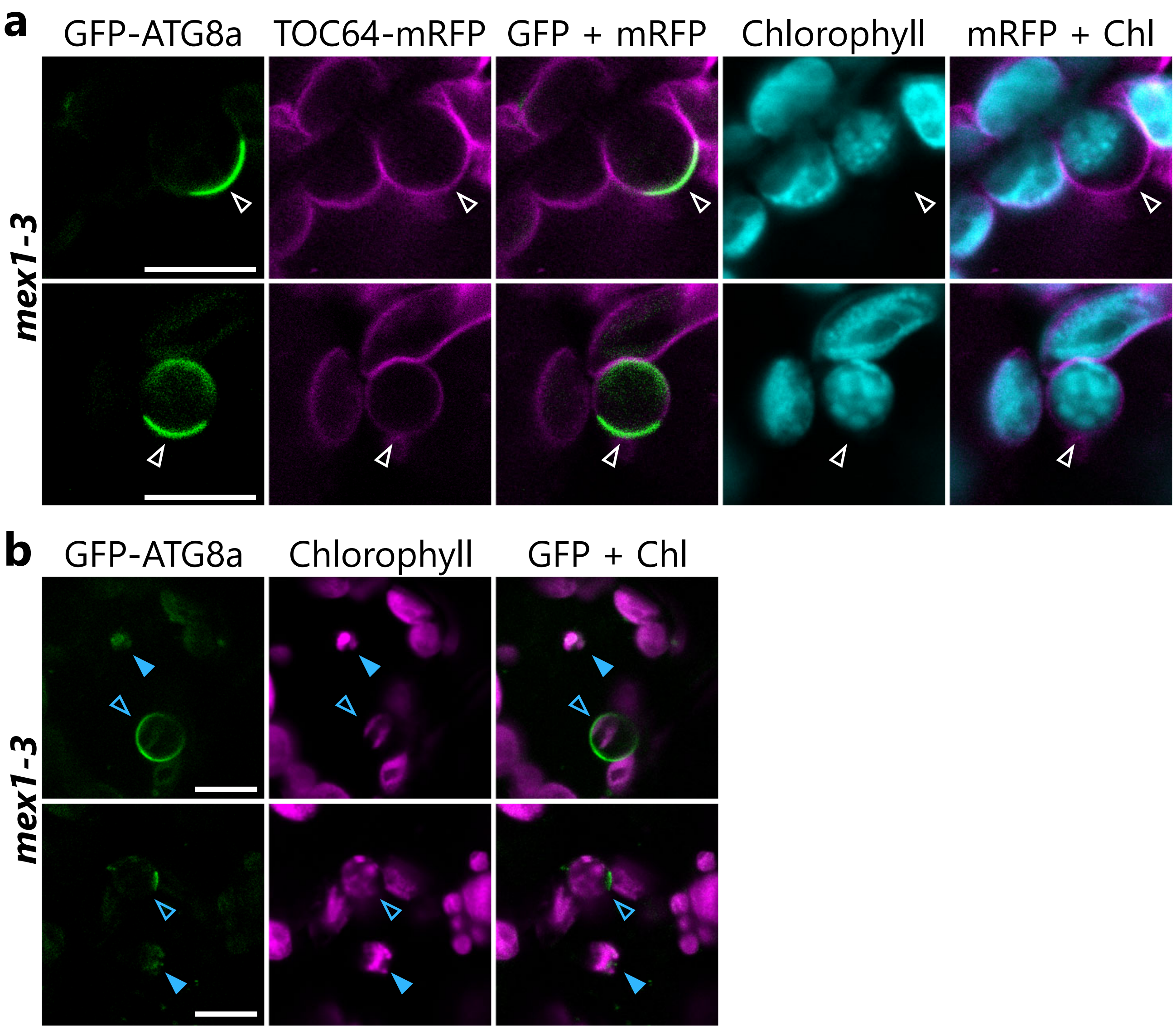

Supplementary Figure S10. Additional observations of the accumulation of GFP-ATG8a on *mex1* chloroplasts.
a) Confocal images of GFP-ATG8a-associated chloroplasts in leaf mesophyll cells from 21-d-old *mex1-3* plants accumulating the autophagosomal membrane marker GFP-ATG8a and the chloroplast outer envelope marker TOC64-mRFP. Green, GFP-ATG8a; magenta, TOC64-mRFP; cyan, chlorophyll fluorescence (Chl). Arrowheads, GFP-ATG8a-associated chloroplasts. Scale bars, 10 μm.
b) Confocal images of GFP-ATG8a-associated chloroplasts in the cytoplasm and vacuole-enclosed chloroplasts being digested in the vacuole in the plants described in (**a**). Green; GFP-ATG8a; magenta, chlorophyll fluorescence (Chl). Open arrowheads, GFP-ATG8a-associated chloroplasts, filled arrowheads, vacuole-enclosed chloroplasts being digested. Scale bars, 10 μm.

Supplementary Figure S11

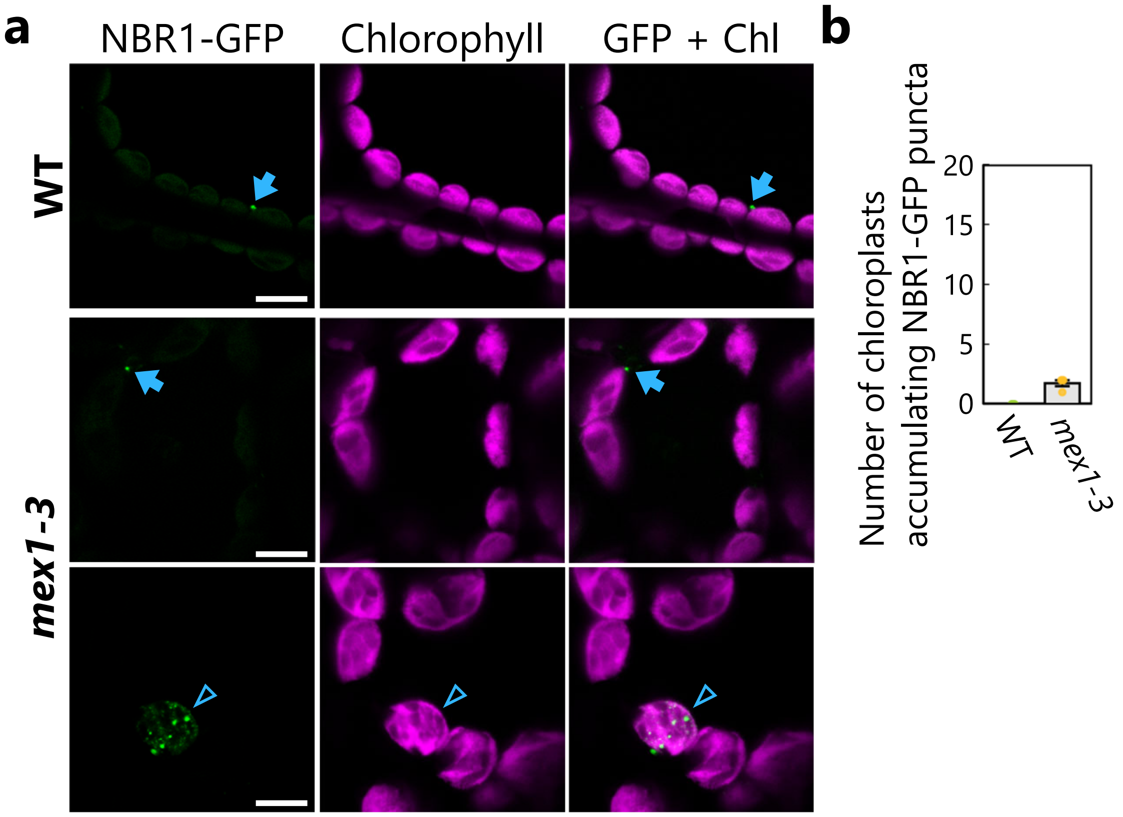

Supplementary Figure S11. A few chloroplasts accumulate dot-like NBR1-GFP signals in *mex1* leaves.

a) Representative confocal images showing NBR1-GFP signals in leaf mesophyll cells from 21-d-old wild type Col-0 (WT) and *mex1-3* plants. Green, NBR1-GFP; magenta, chlorophyll fluorescence (Chl). Arrows, NBR1-GFP-labeled cytoplasmic puncta; arrowheads, a chloroplast accumulating NBR1-GFP puncta. Scale bars, 10 µm.

b) Number of chloroplasts accumulating NBR1-GFP-labeled puncta in a given region based on the observations described in (**a**). The bars indicate means ± SE from four individual plants (n = 4). Dots represent data points from individual plants. The size of the observed region and the scale of the x-axis are the same as in Figure 6b.

Supplementary Figure S12

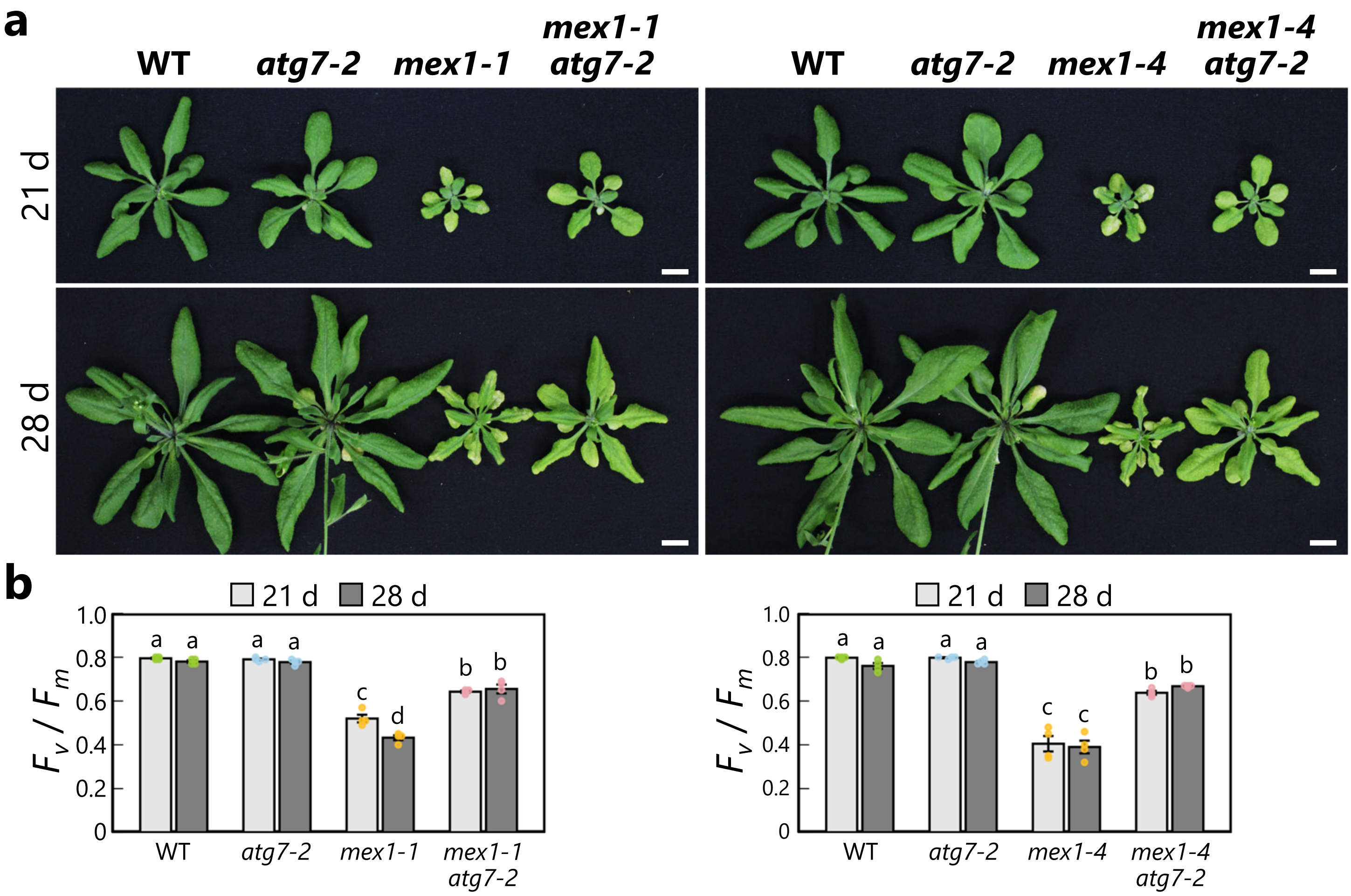

Supplementary Figure S12. The leaf chlorosis observed in *mex1-1* and *mex1-4* alleles is partially suppressed by the loss of ATG7.

a) Representative photographs of wild-type Col-0 (WT), *atg7-2*, *mex1-1*, and *mex1-1 atg7-2* plants (leaf panels) or WT, *atg7-2*, *mex1-4*, and *mex1-4 atg7-2* plants grown for 21 d or 28 d in soil. Scale bars, 10 mm.

b) F_v/F_m values measured from the leaves of the genotypes described in (**a**). Values are means ± SE from four individual plants (n = 4). Dots represent data points from individual plants; different letters denote significant differences based on Tukey's test (P < 0.05).

Supplementary Figure S13

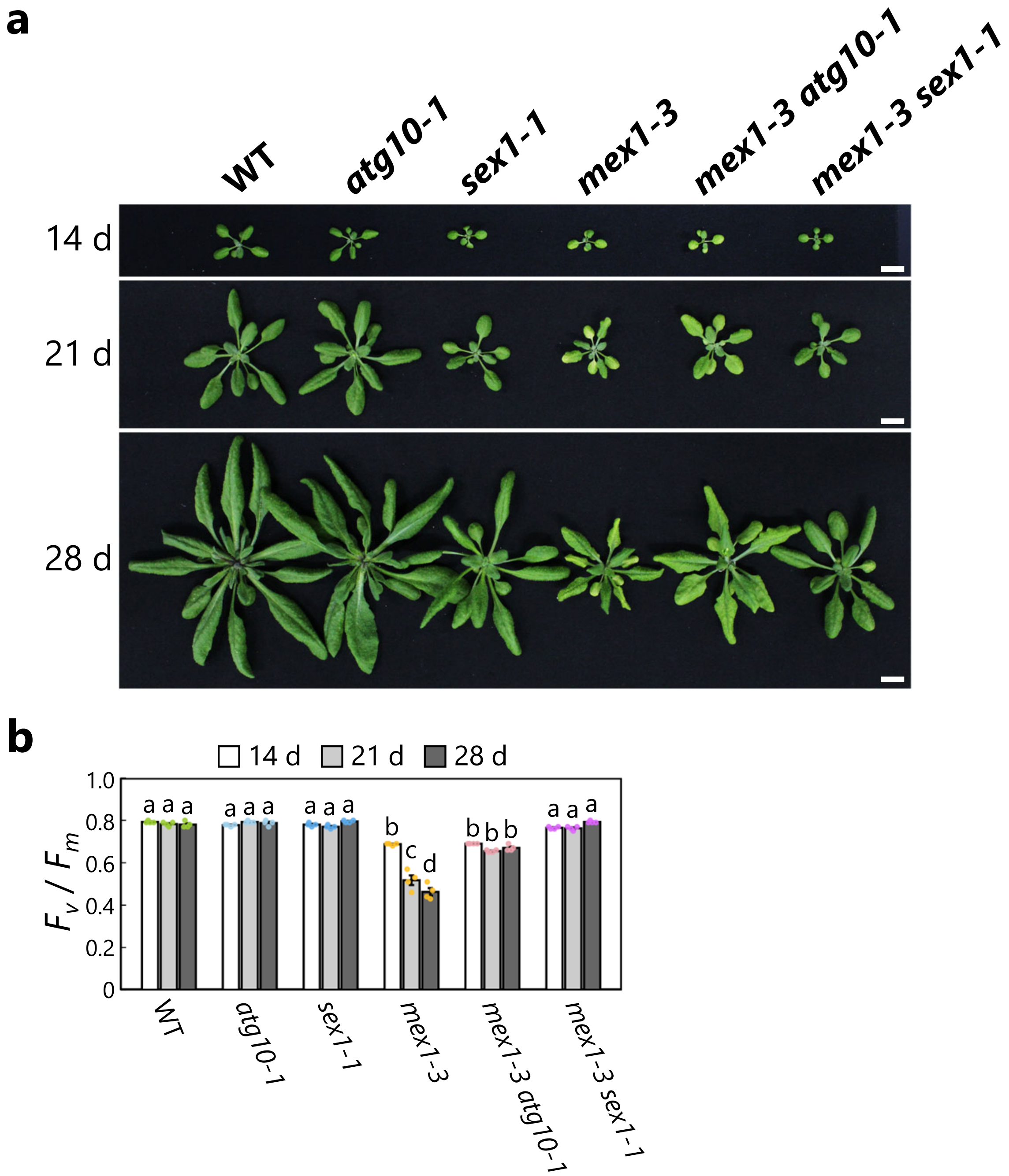

Supplementary Figure S13. The differences in phenotypes between *mex1 atg10* and *mex1 sex1* double mutant plants.
a) Representative photographs of wild-type Col-0 (WT), *atg10-1*, *sex1-1*, *mex1-3*, *mex1-3 atg10-1*, and *mex1-3 sex1-1* plants grown for 14, 21, or 28 d in soil. Scale bars, 10 mm.
b) F_v/F_m values measured from the leaves of the genotypes described in (**a**). Values are means ± SE from four individual plants (n = 4). Dots represent data points from individual plants; different letters denote significant differences based on Tukey's test (P < 0.05).

Supplementary Figure S14

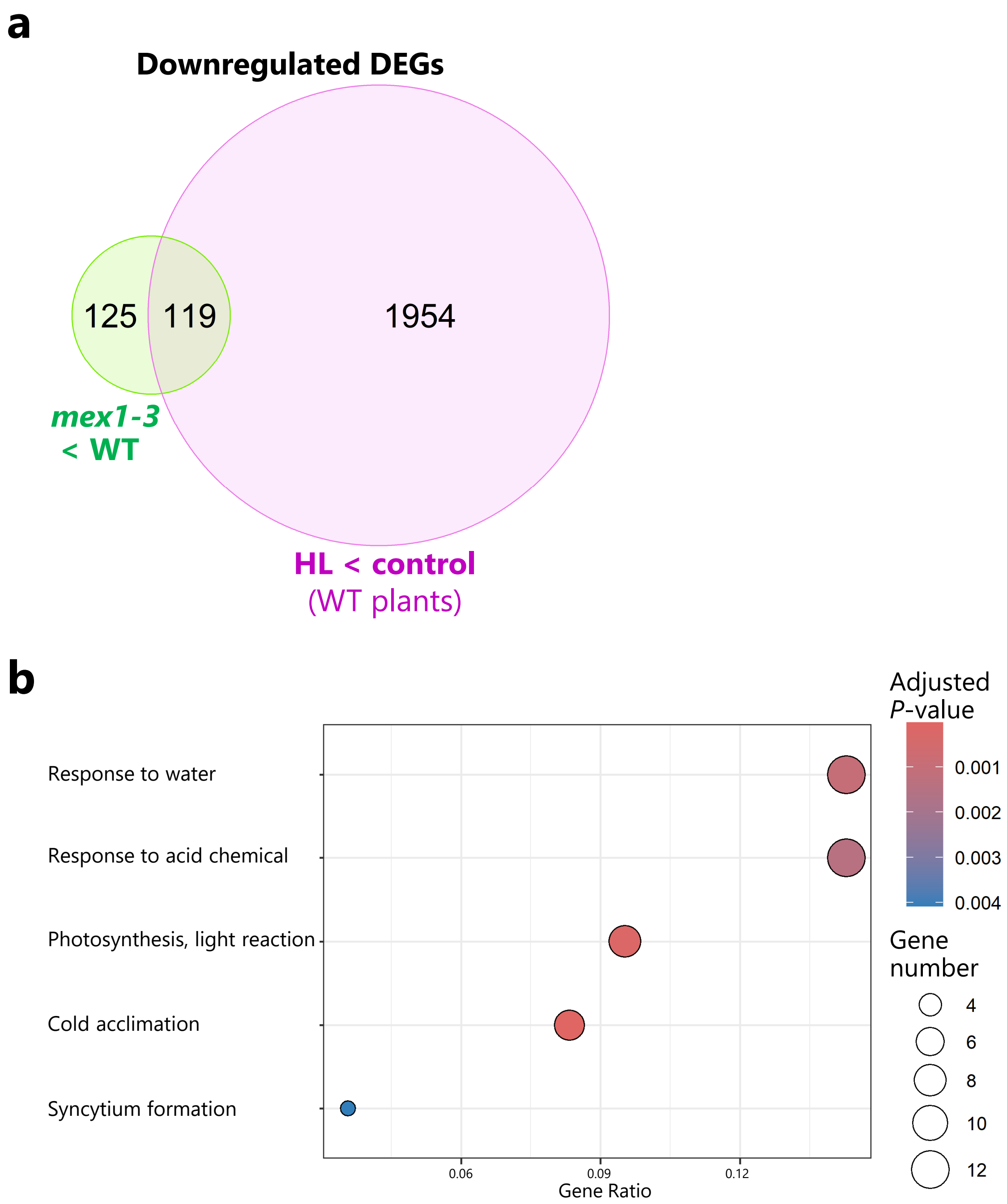

Supplementary Figure S14. Genes downregulated both in *mex1* and following high-light exposure.
a) Venn diagram showing the extent of overlap between the downregulated DEGs in *mex1-3* relative to wild-type Col-0 (WT) and following HL treatment.
b) GO enrichment analysis (biological process) of the downregulated DEGs identified in *mex1-3* and HL-exposed leaves. All enriched GO terms are listed in Supplementary Table S6. Circle size indicates the number of genes enriched in each GO term. Circle colors indicate the adjusted *P*-values, as shown by the color bar.

Supplementary Figure S15

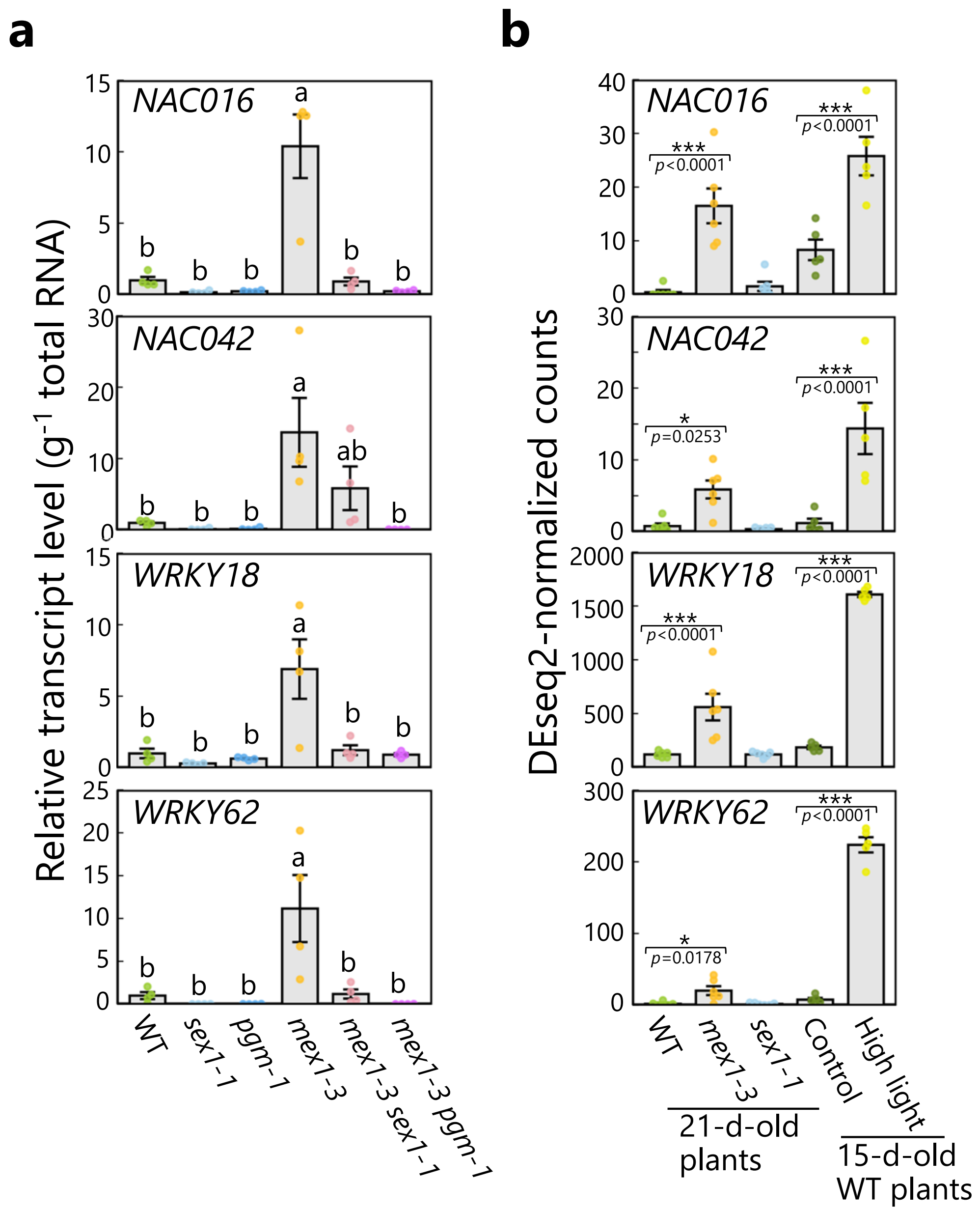

Supplementary Figure S15. RT-qPCR validation of transcript levels from a set of chosen upregulated transcription factor genes in *mex1* and following high-light treatment.

a) Relative transcript levels of *NAC016*, *NAC042*, *WRKY18*, and *WRKY62* measured by RT-qPCR of cDNA samples prepared from total RNA extracted from the leaves of 21-d-old wild-type Col-0 (WT), *sex1-1*, *pgm-1*, *mex1-3*, *mex1-3 sex1-1*, and *mex1-3 pgm-1* plants relative to the values from WT leaves, which were set to 1. The same cDNA samples as in Figure 4 were used. Values are means ± SE from four individual samples (n = 4). Dots represent data points from individual samples; different letters denote significant differences based on Tukey's test ($P < 0.05$)

b) DESeq2-normalized counts for the genes described in (**a**) obtained from the RNA-seq analysis described in Figure 8a. Values are means ± SE from five or six individual samples (n = 5 or 6). Dots represent data points from individual samples; asterisks denote significant differences based on t-test between the indicated samples (*, $P < 0.05$; ***, $P < 0.001$).

Supplementary Figure S16

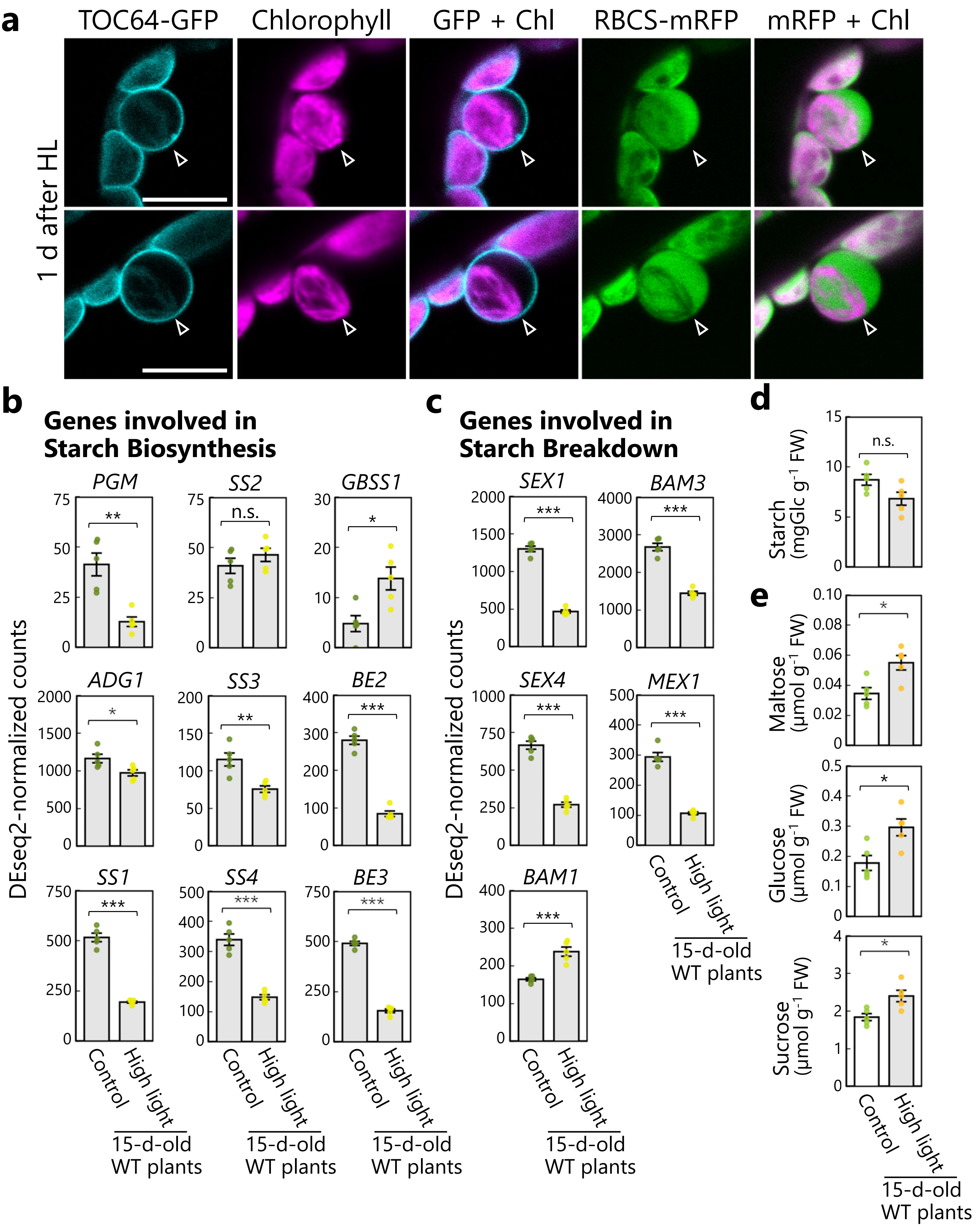

Supplementary Figure S16. Changes in transcript levels of genes involved in starch metabolism and carbohydrate content in leaves exposed to high-light stress.

a) Representative confocal images of swollen chloroplasts caused by 2 h of high-light exposure. Leaves from WT plants accumulating TOC64-GFP and RBCS-mRFP were observed 1 d after HL treatment. Cyan, TOC64-GFP; magenta, chlorophyll fluorescence (Chl); green, RBCS-mRFP. Arrowheads, swollen chloroplasts. Scale bars, 10 µm.

b, c) DESeq2-normalized counts for genes involved in starch biosynthesis (**b**) and breakdown (**c**) in the leaves of 15-d-old plants 1 d after high-light exposure or untreated control plants obtained from the RNA-seq analysis described in Figure 8a.

d, e) Starch (**d**) and sugar (**e**) contents in the samples described in (**b**, **c**).

In each plot, values are means ± SE from five individual samples (n = 5). Dots represent data points from individual samples; asterisks denote significant differences based on t-test (n.s., not significant; *, P < 0.05; **, P < 0.01; ***, P < 0.001).
